# Computational analysis reveals histotype-dependent molecular profile and actionable mutation effects across cancers

**DOI:** 10.1186/s13073-018-0591-9

**Published:** 2018-11-15

**Authors:** Daniel Heim, Grégoire Montavon, Peter Hufnagl, Klaus-Robert Müller, Frederick Klauschen

**Affiliations:** 1 Institute of Pathology, Charité - Universitätsmedizin Berlin, corporate member of Freie Universität Berlin, Humboldt-Universität zu Berlin and Berlin Institute of Health, Berlin, Germany; 2 Department of Electrical Engineering and Computer Science, Technische Universität Berlin, Marchstr. 23, 10587 Berlin, Germany; 3 Department of Computer Science, Machine Learning Group, Berlin Institute of Technology, Marchstr. 23, 10587 Berlin, Germany; 4 Bernstein Focus Neurotechnology, Berlin, Germany; 5 Max Planck Institute for Informatics, Stuhlsatzenhausweg, 66123 Saarbrücken, Germany; 6 Department of Brain and Cognitive Engineering, Korea University, Anam-dong, Seongbuk-gu, Seoul, 02841 South Korea; 7 German Cancer Consortium (DKTK), Partner Site Berlin, Berlin, Germany; 8 German Cancer Research Center (DKFZ), Heidelberg, Germany

**Keywords:** Cancer, Genomics, Pan-cancer analysis, Proteomics, Targeted cancer therapy

## Abstract

**Background:**

Comprehensive mutational profiling data now available on all major cancers have led to proposals of novel molecular tumor classifications that modify or replace the established organ- and tissue-based tumor typing. The rationale behind such molecular reclassifications is that genetic alterations underlying cancer pathology predict response to therapy and may therefore offer a more precise view on cancer than histology. The use of individual actionable mutations to select cancers for treatment across histotypes is already being tested in the so-called basket trials with variable success rates. Here, we present a computational approach that facilitates the systematic analysis of the histological context dependency of mutational effects by integrating genomic and proteomic tumor profiles across cancers.

**Methods:**

To determine effects of oncogenic mutations on protein profiles, we used the energy distance, which compares the Euclidean distances of protein profiles in tumors with an oncogenic mutation (inner distance) to that in tumors without the mutation (outer distance) and performed Monte Carlo simulations for the significance analysis. Finally, the proteins were ranked by their contribution to profile differences to identify proteins characteristic of oncogenic mutation effects across cancers.

**Results:**

We apply our approach to four current proposals of molecular tumor classifications and major therapeutically relevant actionable genes. All 12 actionable genes evaluated show effects on the protein level in the corresponding tumor type and showed additional mutation-related protein profiles in 21 tumor types. Moreover, our analysis identifies consistent cross-cancer effects for 4 genes (FGFR1, ERRB2, IDH1, KRAS/NRAS) in 14 tumor types. We further use cell line drug response data to validate our findings.

**Conclusions:**

This computational approach can be used to identify mutational signatures that have protein-level effects and can therefore contribute to preclinical in silico tests of the efficacy of molecular classifications as well as the druggability of individual mutations. It thus supports the identification of novel targeted therapies effective across cancers and guides efficient basket trial designs.

**Electronic supplementary material:**

The online version of this article (10.1186/s13073-018-0591-9) contains supplementary material, which is available to authorized users.

## Background

Next-generation sequencing has facilitated comprehensive mutational profiling of all major cancers and has led to the discovery of oncogenic driver mutations, many of which can be targeted therapeutically [1–3]. Following the conventional organ- and tissue-based WHO classification of tumors and standard clinical trial design, precision therapies targeting these driver mutations are usually evaluated for a specific cancer type. However, sequencing data has shown that actionable mutations, albeit with different frequencies, occur across cancers, which has raised the question about histotype-independent therapies and novel ways of tumor classifications no longer relying on histology but on genetic profiles. Recent studies propose such molecular tumor classifications, which extend or even replace the histology-based tumor typing as implemented by the World Health Organization (WHO) [4, 5]. Although different, these approaches share the common idea that molecular (mutational) profiles govern tumor pathology and should therefore replace histotyping in diagnostics and therapy selection [6]. That targeted therapies against the same single molecular alteration can be effective across cancers, as shown, for instance, by the efficacy of anti-Her2 therapy in both gastric and breast cancers [7, 8] or the clinical benefit from inhibition of mutated cKIT in gastrointestinal stromal tumors (GIST) and melanoma or mastocytosis [9, 10]. However, the fact that inhibition of BRAF mutated at V600 is effective in melanoma but not in colorectal cancer [1, 11, 12] is a prominent example against the general transferability of knowledge on a single actionable mutation from one histological tumor type to another. This observation is corroborated by recent basket trials that point to histology as an important predictor of response to targeted therapy against actionable mutations [13, 14].

The reasons for the variable therapeutic utility of genetic aberrations across cancers is likely due to the complex molecular “background” observed in many tumors. Using mutational profiles or just single genetic aberrations, as is the case in the current basket trials, is unlikely to cover the full scope of (tissue-specific) molecular effects including epigenetic mechanisms and downstream regulation such as post-translational modifications. However, it would be highly desirable for therapy selection in individual patients and clinical trial design to predict genes with similar functional effects across histotypes.

**Fig. 1:**
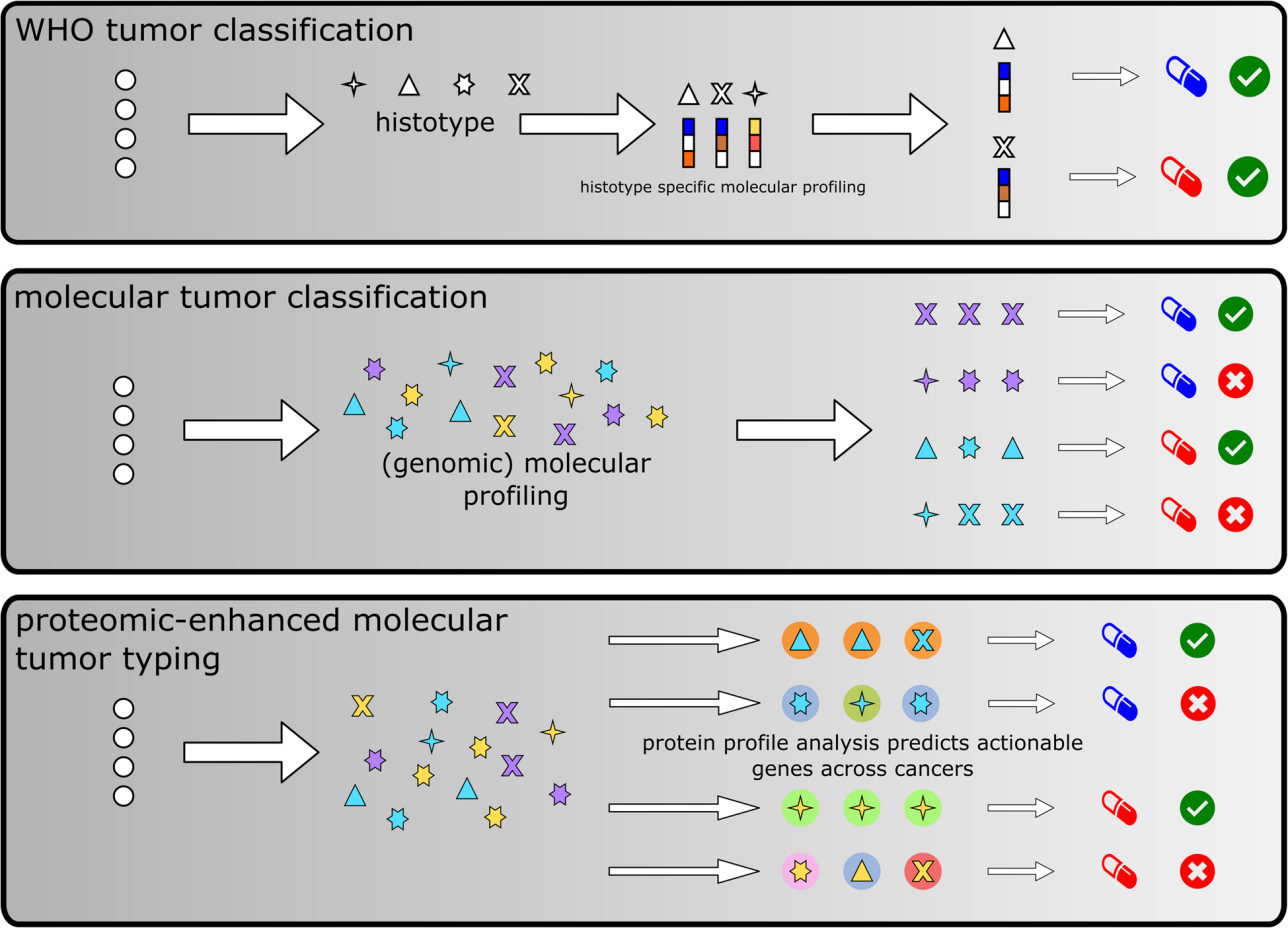
Graphical abstract/flow diagram of study. Top: In the WHO classification, tumors are typed by their histomorphological properties which are refined by additional molecular markers based on which targeted therapies are selected if actionable mutations are found. Middle: Novel molecular tumor classifications propose to ignore histological properties and fully rely on comprehensive molecular profiling dominated by genomic techniques based on which targeted therapies are selected, which are, however, not always effective if histotypes are ignored. Bottom: The approach we propose complements genomic profiling by the integration of proteomic data to estimate the functional relevance of mutations and predict the efficacy of targeted therapies. Our results show that actionable mutations are associated with distinct proteomic profiles and are indicative of drug response in cell line data

To this end, we developed a computational approach that integrates genomic and proteomic data from 3590 tumors to analyze the impact of genetic aberrations on protein profiles and gauge the functional effects of mutational profiles across 32 different cancers. We apply our approach to evaluate the (functional) relevance of the abovementioned molecular tumor classifications and systematically analyze the effects of all major driver mutations on protein profiles across all major cancers. Figure 1 provides an overview of our approach and compares it to traditional tumor classifications.

## Methods

### Genetic data

Two TCGA data types were used: protein expression and somatic mutations. Data of all diseases available without limitation was used. For somatic mutation data, we used data processed with Oncotator [15]. TCGA data was downloaded from Broad Institute [16] via GDAC firehose http download. For pan-organ protein expression analysis, we used TCPA data [17] (https://www.tcpaportal.org/tcpa/; TCGA-PANCAN16-RBN.csv).

### Batch removal

To remove possible TCGA batch effects on protein expressions influencing our analysis, we excluded 21 batches with a total of 393 TCGA cases (details given in Additional file 1: Table S1). To identify problematic batches, we used the R package of mbatch (http://bioinformatics.mdanderson.org/tcgambatch/). For each histological tumor type, we sequentially removed batches until the value of the Dispersion Separability Criterion (DSC) was below 0.3 and the corresponding *p* value was below 0.05. The batch to remove next was chosen by iteratively removing one of the current batches, calculating the DSC value for a remaining set of batches, and adding the batch again. The batch with the lowest DSC for the remaining batches after its removal was chosen.

### Tumor classifications

Tumor classifications proposed by Ciriello et al. [4] and Hoadley et al. [5] were investigated. We also tested a classification we published earlier based on the definition of a nearest mutational neighbor by Heim et al. [18]. The fourth classification we analyzed is based on the genes TP53, TTN, and BRAF. These three genes were chosen after a principal component analysis of mutational profiles showed they identify the coarse cluster structure of the data when mutational profiles are represented as two-dimensional vectors (more details given in Additional file 1, section “Genetic complexity reduction”). Additionally, classes comprising of cases with typical alterations in actionable genes such as BRAF V600 were evaluated. The classifications based on actionable genes are binary; this means for each actionable gene, the classification has two classes—one class is comprised of cases having that actionable mutation the other of those without. All analyses described in this paper were performed for each classification separately.

### Discriminability analysis

To test whether there are differences in protein expression between cases of different classes from the same tumor, we computed the energy distance of the two groups composed by those cases. Energy distance measures the homogeneity of protein expression of group A (for example, skin cancer melanoma BRAF V600E-positive cases) and group B (skin cancer melanoma BRAF V600E-negative cases) separately and compares it to the homogeneity of cases from A to B taken together. A smaller (negative) energy distance indicates the protein expressions of the cases from the two groups are discriminable. Let ${c}_1^{1,A}$ to ${c}_i^{1,A}$ be the group *g*^1, *A*^ of *i* cases with histological tumor type A assigned to class 1, ${c}_1^{2,\kern0.5em A}$ to ${c}_j^{2,A}$ be the group *g*^2, *A*^ of *j* cases with histological tumor type A assigned to class 2, and let *d*(*c*_*x*_, *c*_*y*_) be the Euclidean distance of two cases in protein space, than the energy distance *de*_1*A*, 2*A*_ of the two groups *g*^1, *A*^ and *g*^2, *A*^ is defined as:

$$ {de}_{1A,2A}=\sum \limits_{k=1}^{i-1}\sum \limits_{l=k+1}^id\left({c}_k^{1,A},{c}_l^{1,A}\right)+\sum \limits_{k=1}^{j-1}\sum \limits_{l=k+1}^jd\left({c}_k^{2,A},{c}_l^{2,A}\right)-2\ast \sum \limits_{k=1}^i\sum \limits_{l=1}^jd\left({c}_k^{1,A},{c}_l^{2,A}\right) $$

To test whether the distance between two groups is significant or within random range, we ran Monte Carlo simulations (1 million runs) to determine the *p* value corresponding to the measured distance. In each run, we calculated the energy distance of two groups, which were randomly created from the same cases as originally used and having the same sizes *i* and *j* as the original groups. The frequency of the two random groups having a lower or equal energy distance (stronger or equal differences in protein expressions) than the groups based on the original genetic classes yields the *p* value. Benjamini-Hochberg procedure (*p* values given in the text are unadjusted) and *p* value thresholding (*p* < 0.05) were used to find the pairs of genetic classes with significantly different protein expressions for each histological tumor type. This results in a list of pairs of groups (a pair of groups are two groups of cases from the same histotype with different genetic classes) with significant differences in protein expressions between the two groups. As a trade-off between the reliability of results and investigating as many classes as possible, we compared only groups with five or more cases.

### Determine characteristic proteins

For each pair of groups with significant differences in protein expression, we also calculated the contribution of each protein to the total energy distance of the two groups. Therefore, the energy distance of the two groups is calculated as described above, with the modification that the distance of two cases is not given by their Euclidean distance in protein space but by the difference in expressions for a specific protein only. Calculated for each protein separately, the contribution of each protein to the distance is known. As a result, we were able to filter for those proteins that have the strongest effect on the discriminability of the two different groups. The proteins with the strongest contribution to the difference of the two groups are those with the most negative contribution values. Proteins with a negative contribution value are considered to be characteristic proteins if the absolute value of the contribution of the protein is higher than the highest positive contribution value among all proteins. We chose this dynamic threshold as it can be considered as an estimate for the maximum noise of the contribution signals. Characteristic proteins are then divided into two sets by calculating their mean values among the cases of the two groups. The proteins with higher mean values in group A are considered to have significantly increased expression values in group A cases compared to group B cases and vice versa. We chose this test for characteristic proteins over, for example, the *U* test because it puts emphasis on the proteins with larger differences rather than proteins with smaller yet significant differences between the two groups of cases.

### Definition of cross-cancer effects

To address the question of how mutational differences between two classes affect protein expressions in more than one histotype in the same way, we performed a cross-cancer effect analysis. We therefore searched the list of discriminable group pairs for group pairs with the same classes but different histological tumor types. The result would be two group pairs *pA* = (*g*^1, *A*^, *g*^2, *A*^) and *pB* = (*g*^1, *B*^, *g*^2, *B*^). Associated with each pair of groups are two sets of characteristic proteins. Set ${s}_1^A$ is the set of characteristic proteins for *pA* that are increased for class 1, set ${s}_2^A$ is the set of characteristic proteins for *pA* that are increased for class 2, set ${s}_1^B$ is the set of characteristic proteins for *pB* that are increased for class 1, and set ${s}_2^B$ is the set of characteristic proteins for *pB* that are increased for class 2. We then compute the number of intersecting characteristic proteins by $ni=\left|{s}_1^A\cap {s}_1^B\right|+\left|{s}_2^A\cap {s}_2^B\right|$ and use one-tailed Fisher’s exact test to determine whether the number of intersecting characteristic proteins is significant relatively to the number of characteristic proteins of both groups and the total number of proteins used (120). The *p* value for all group and histological tumor type pairs were also false positive corrected with the Benjamini-Hochberg procedure.

## Results

### Proteomic profiles are more histotype-specific than genomic profiles

The attempts to propose novel molecular tumor classifications are mainly based on the observation that genomic profiles show substantial similarities across histological tumor types and are often inconsistent with histology [4, 18]. Because this study aims at evaluating the functional relevance of these molecular classifications based on proteomic profiles, we first studied whether the observed inconsistencies between genomic and histological typing also exist on the level of proteins by re-applying the analysis presented by Heim et al. [18] to corresponding reverse-phase protein array data available through The Cancer Protein Atlas (TCPA) [17].

**Fig. 2:**
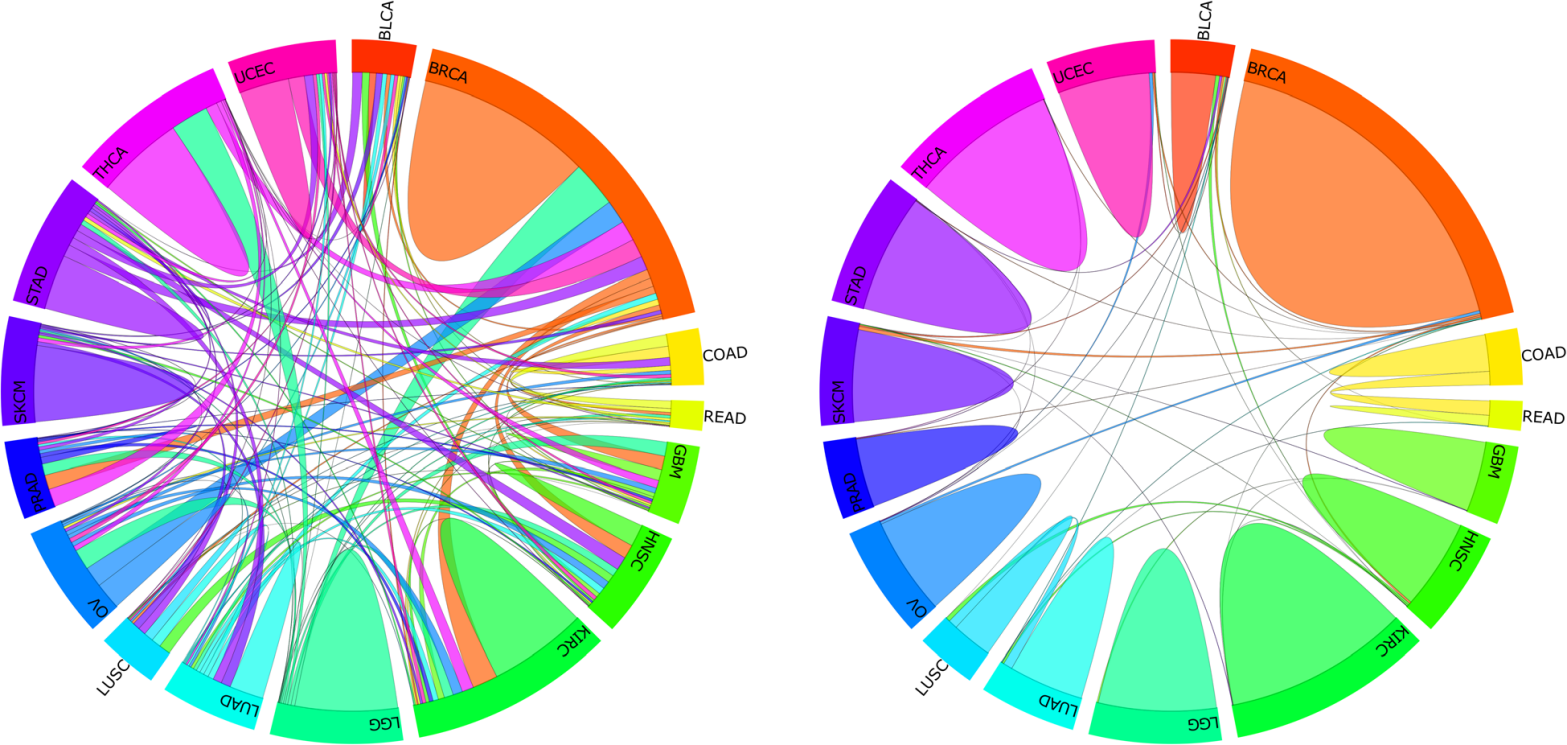
Mutational and proteomic cross-cancer similarities. Chord diagrams show the cross-cancer similarities for somatic mutations (left) and protein profiles (right) by computing for each case the closest molecular neighbor among all 3590 tumors for which mutational and proteomic data were available. Chords connecting two histological tumor types indicate the number of tumors of a certain cancer that are—on the level of mutations or protein profiles—more similar to tumors of the other type than to their own, indicating a disagreement of molecular and histological type. Hill-like structures, on the other hand, indicate the amount of cases where molecular and histological classes are identical. It is obvious that a substantial disagreement exists only for mutational profiles (similar results for copy number variation,  Additional file 1: Figure S4) showing agreement between mutational and histological tumor types in only 45% of the cases, whereas protein profiles are consistent with histological tumor types in over 94%

Our analysis showed that mutational and histological tumor types agreed for only 45% of the 3590 cases, for which both protein and mutational data were available (47% if combining colon and rectal cancer as a single histotype). For protein profiles, the analysis demonstrates a consistency with histotypes in 94% of the cases (95.6% if colon and rectal cancer combined, Fig. 2). The only relevant cross-cancer similarities on the protein level exist for colon and rectal cancer (1.6% of all cases) and, moreover, lung squamous and adenocarcinoma (1%), which arise in the same organ.

To further examine the proteogenomic relations and to test if distinct mutations converge on similar protein profiles, we performed a gene set enrichment analysis that evaluated if the used gene set contains a significant number of genes corresponding to differentially expressed proteins. We found such relations in 24 out of 76 pair-wise molecular class comparisons, all of which were histotype-dependent (for details about the method, see Additional file 1, section “The relation between mutational and protein profiles,” and for results, see Additional file 2: Table S2).

Moreover, we tested if the fact that TCGA/TCPA offers only a limited panel of less than 200 proteins may introduce a bias when comparing cross-cancer similarities between genomic and proteomic data. Also, evaluating functional groups of genes may show different patterns of genetic profiles. To address these issues, we first re-performed our similarity analysis for only the genes with corresponding proteins in the TCPA/RPPA data. Second, we assigned genes to the c6 gene set (oncogenic signaling) from MSigDB (Broad Institute). These controls showed that apart from minor quantitative differences, the overall pattern of substantial cross-cancer similarities is consistent between both approaches and our original findings shown in Fig. 2 (for details, see Additional file 1, section “Cross-cancer similarities for different gene sets”).

In summary, while substantial genetic cross-cancer similarities exist, our analysis points to a pronounced organ-type specificity of the observed protein profiles. At this point, it is unclear whether the reason for this inconsistency between genetic and protein profiles is the differential translation of genetic profiles into protein levels in different cancer types, or organ- and tissue-specific protein base levels that are modulated by mutations—or a combination of both.

### Histotype specificity of genetic classifications and pan-cancer effects

Based on the findings that global genetic cross-cancer similarities are not reflected in corresponding protein pattern similarities, we evaluated to what extent tumor classifications based on molecular alterations are impacting protein profiles. We systematically compared if, and which, genetic classes affect proteins (i.e., which classes are discriminable on the level of protein profiles) for 3 molecular cancer classification approaches [4, 5, 18] and a computational approach based on reducing genetic complexity for 30 different histological tumor types. Subsequent to the identification of molecular classes with impact on the protein level as an indicator of functional relevance within each histotype, we also compared the molecular class discriminability on the protein level across histological tumor types to determine which of the molecular classes found to be functionally relevant in one histotype, are relevant also in another. To this end, the following aspects are addressed for each classification.

First, we provide a descriptive comparison between genetic and histological classifications by evaluating the distribution of genetic classes across the different histological classes.

Secondly, we introduce the classification effectivity score (CES) which indicates to what extent different mutational classes can be discriminated by their protein profiles assuming that classifications are more effective and thus clinically relevant if their (genetic) classes are visible also on the protein level. The CES integrates the results of the different subtests (a subtest is the pair-wise comparison of two genetic classes with respect to their protein profiles) into one score to describe the accuracy of the classification with which individual classes can be discriminated by protein profiles (i.e., the percentage of evaluated class vs. class subtests where the protein profiles were found to be significantly different from each other). The CES can be also evaluated class-wise (only subtests using a specific class are evaluated) to describe to what extent protein profiles of the specific class differ from those of all other classes. In analogy to this, the histotype-wise CES evaluates all subtests for a specific histotype.

Thirdly, in addition to the global CES, we define the discriminability score s_dis_ for pairwise class comparisons. The discriminability score measures to what extent protein profiles of histological tumor type X differ between class A and class B (as s_dis_ the describes the difference in protein levels of two classes, the score is more negative the more distinct the profiles of the two sets are). For those subtests, we also identify those proteins for which the profiles deviate most between two classes.

Finally, we analyze the cross-cancer protein profile effects for each classification. A cross-cancer effect is reported when two genetic classes can be distinguished from each other in protein profiles in at least two histological tumor types and if protein effect directionality is similar across histotypes.

Overall, the four tested molecular classifications define classes that are associated with distinct protein profiles in some tumor types. However, protein profiles are affected similarly across tumor types for only two class pairs, and for most classes, protein profile discriminability is dependent on tumor type.

**Fig. 3:**
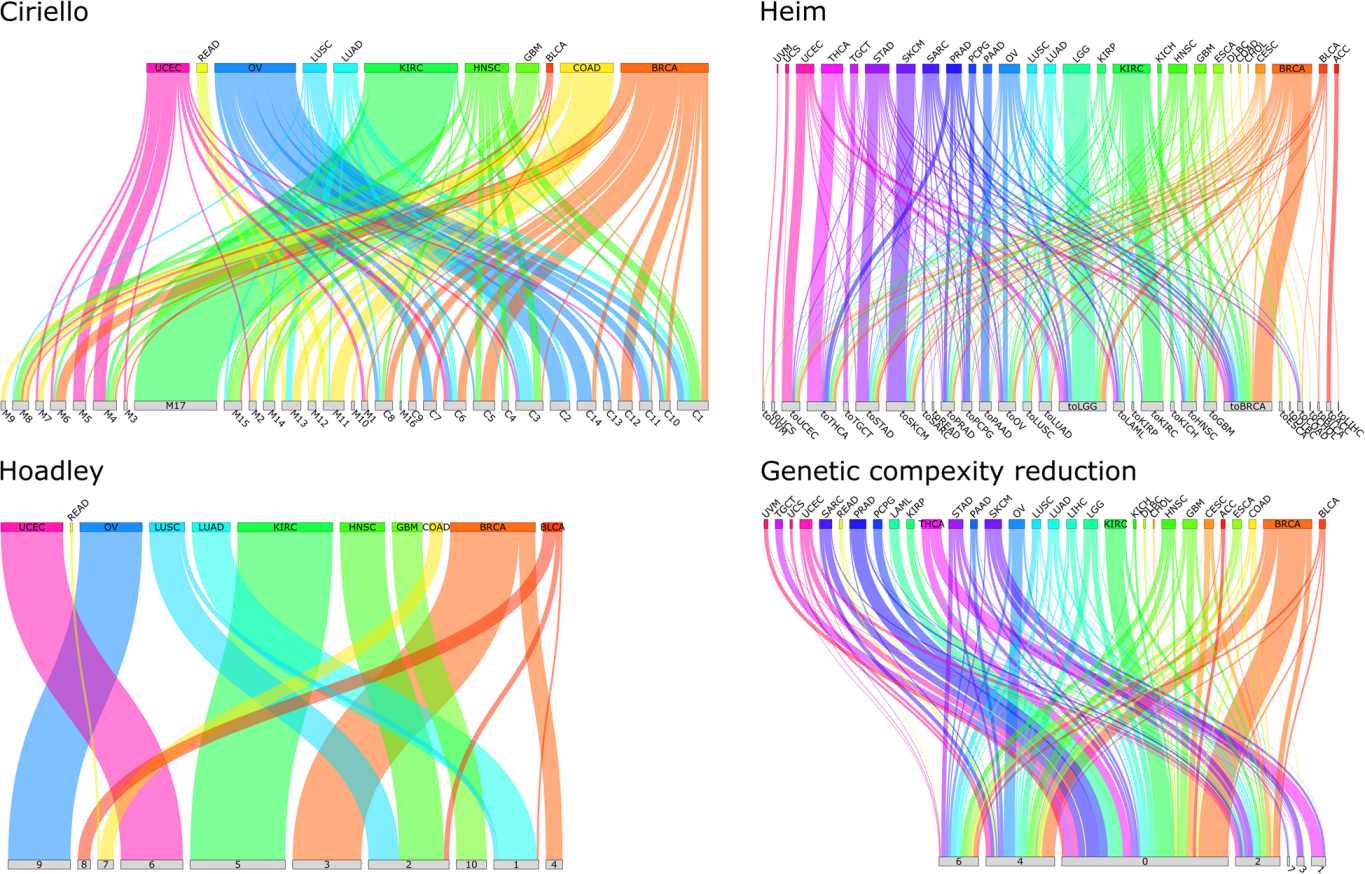
Relations of molecular classes and histological tumor types in each classification. The diagrams show the number of cases from each histotype (top) assigned to the molecular classes (bottom). The molecular classification by Hoadley et al. is largely consistent with histotyping as a molecular class contains cases from only 1.5 histotypes on average. Ciriello et al. shows 2.5 histotypes per molecular class, whereas the classifications based on Heim et al. and genetic complexity reduction are substantially more distinct from histotyping as a molecular class is comprised of cases of 5.4 respectively 10.75 different histological tumor types

In the proposal by Ciriello et al. [4], tumors are classified by the presence of somatic mutations and copy number alterations in cancer-related pathways. Molecular classes are composed of, on average, 2.5 different histotypes (range 2 to 6), and histotypes are assigned to 7.2 different molecular classes on average (range 2 to 15). For details about histotype and molecular class congruence, also see Fig. 3.

**Fig. 4:**
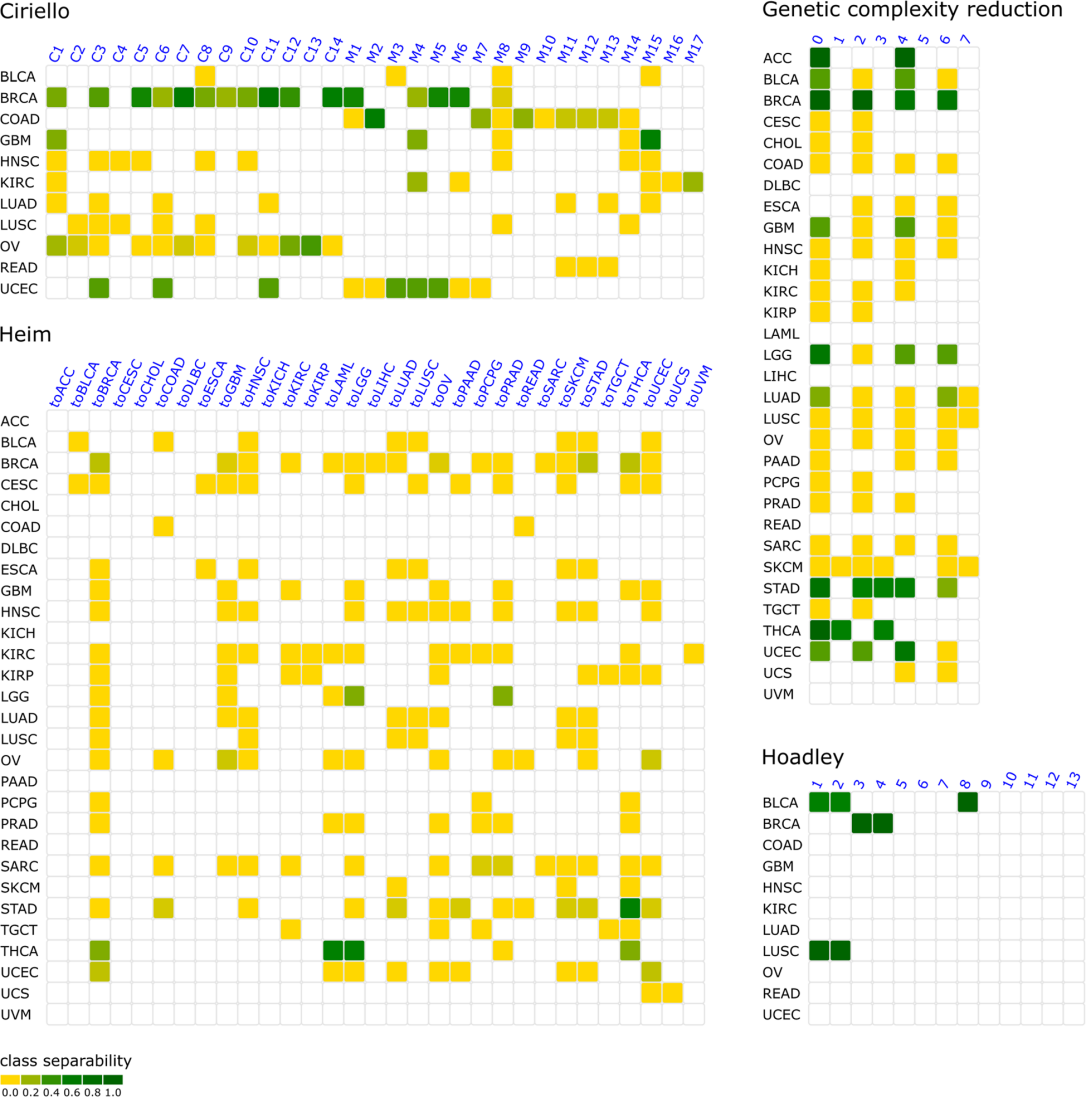
Reproducibility of molecular tumor classes on the level of protein profiles. The heatmaps show for each molecular tumor classification and all histotypes the degree with which a molecular class is distinguishable from other classes based on protein profiles. Blank matrix entries indicate fewer than five cases which were excluded from the analysis. In the classification by Ciriello et al., gynecological cancers (breast, uterine and ovarian) show highest protein-level effects of genetic classes, whereas the molecular classes based on Heim et al. have less impact on proteins overall. The genetic complexity reduction classification has relatively distinct protein profiles for histological tumor types. Hoadley et al. propose classes well discriminable by protein profile (however, due to the high agreement with histotypes, only a few classes contain sufficient cases to evaluate protein level effects)

The global classification effectivity score (CES) is 17%. The highest effectivity score for a specific histotype is found for breast cancer with 36.6%. The molecular classes, which show the highest classification effectivity in breast cancer, are C11 (66%) and C7 (60%). Yet, both classes are separable from other classes in hardly any other histological type (C11: CES = 11%, C7: CES = 9% over all other histotypes). For histological tumor types other than breast cancer, the mean classification effectivity score is 10%. Highest histotype-specific classification effectivity scores apart from breast cancer are found for colorectal adenocarcinoma (CES = 13%), ovarian cancer (CES = 9%), and endometrial carcinoma (CES = 20%), with CES = 36% for breast cancer being the highest score, followed by endometrial carcinoma with 20% and 10% for other histotypes. Our results indicate that the Ciriello classification is to some extent focused on breast cancer. An overview on class and histotype classification effectivity scores is also given in Fig. 4.

The highest molecular class discriminability score is that within breast cancer cases for classes C11 and M1 with a score of s_dis_ = − 6.38 (*p* = 2.1e−3, based on a Monte Carlo simulation with 1e6 random class assignment runs resulting in an average discriminability score s_rand_ = − 3.5e−4). Comparing the protein expression of the two classes shows that the average expression of Cyclin_B1 is significantly increased for class C11 cases whereas PR, GATA3, ER-alpha, Bcl-2, and Caveolin-1 expression values are significantly higher for M1 cases. Interestingly, high Cyclin-B1 and low PR expression indicate a (more aggressive) basal subtype of breast cancer in the PAM50 profile which indicates that the proteomic profiles at least partially correlate with gene expression-based subtyping although this cannot be comprehensively tested here due to the small panel of available proteins.

The least pronounced but still significant class discriminability is achieved for classes C12 and C5 in breast cancer (s_dis_ = − 0.31, *p* = 4.4e−3; s_rand_ = − 8.0e−5). Class C12 has significantly increased mean expression/phosphorylation for Syk, p70S6K, HER2_pY1248, EGFR_pY1068, and HER2; class C5 is enriched for higher expression of PR, ER-alpha, GATA3, and Cyclin_D1.

To search for consistent effects of genetic alterations on protein profiles not just within a histological tumor type but across tumor types, we evaluate the overlap of proteins with altered expressions/phosphorylation for all molecular classes that are discriminable in more than one histotype. Pairs of molecular classes that are discriminable in more than one histological tumor type and therefore candidates for such an effect are, in principle, classes C11 and M4 in breast cancer and endometrial carcinoma, classes C11 and M5 in breast cancer and endometrial carcinoma, and classes C3 and M5 in breast cancer and endometrial carcinoma. However, no significant intersection of characteristic proteins between breast cancer and endometrial carcinoma can be found for any pair of classes, and therefore, no cross-cancer effects are present for the Ciriello classification (complete results are available in the Additional file 3).

The classification proposed by Hoadley et al. [5] consists of 13 different classes based on comprehensive proteogenomic information. A molecular class comprises, on average, 1.5 different histological types (min 1; max 3). Classes 3, 4, 5, 6, 8, 9, and 10 comprise only of cases of a single histotype. Cases from one histotype are assigned to 1.3 different classes on average (range 1 to 3). Compared to Ciriello et al., the Hoadley proposal has a substantial overlap with the histological tumor types. In particular, five or more cases were assigned to more than one molecular class only for lung squamous cell carcinoma, breast cancer, and urothelial bladder carcinoma. Because subtests are conducted for pairs of molecular classes within the same histotype, only five subtests can be performed. Four of those five class pairs show significant differences in protein expression resulting in an overall classification effectivity score (CES) of 80% (urothelial bladder carcinoma 66%, breast cancer 100%, and lung squamous cell cancer 100%). The discriminability score is s_dis_ = − 4.24 (*p* = 0.0; s_rand_ = 8.1e−5) in breast cancer for molecular classes 3 and 4. The proteins with significant expression differences responsible for discriminability of classes 3 and 4 are INPP4B, ER-alpha, GATA3, PR, AR, Bcl-2, and Cyclin_B1. Significant differences in protein expression (s_dis_ = − 0.58; *p* = 1.6e−5; s_rand_ = 3.5e−5) are also found for lung squamous cell carcinoma cases assigned to molecular classes 1 and 2. For urothelial bladder carcinoma, classes 1 and 8 (s_dis_ = − 1.34; *p* = 7.0e−5; s_rand_ = 2.6e−5) are discriminable as well as classes 2 and 8 (s_dis_ = − 1.41; *p* = 0.0; s_rand_ = 3.2e−5). Because no pairs of molecular classes are discriminable in more than one histological tumor type, no cross-cancer effects can be found.

Heim et al. [15] compute mutational profile similarity classes across all tumors based on somatic mutations. For example, a breast cancer case with a mutational profile that is most similar to an ovarian carcinoma case is assigned the class toOv. On average, cases from one histotype are assigned to 5.4 different classes (min 1, max 16), and one class consists of cases from 5.1 different histological tumor types. As reported in [18] for each “to-histotype” class, the majority of assigned cases belongs to this tumor type (toBRCA consists of 50% breast cancer cases for instance).

With CES = 2%, the overall classification effectivity score of this classification is the lowest among all tested classifications indicating that global comparisons based on somatic mutations only are not effective in classifying tumors in a meaningful way if the available protein profiles are considered relevant. Histotype-specific classification effectivity scores are highest for breast cancer (2.5%), low-grade glioma (10%), gastric cancer (7%), and thyroid carcinoma (30%). Of all classes in this classification, toTHCA (the class consisting of cases that have a mutation profile most similar to thyroid carcinoma) had the highest classification effectivity score of 8%.

For this classification, class discriminability s_dis_ is highest between class toLGG (cases that are most similar to low-grade glioma cases by their mutation profile) and class toPRAD for low-grade glioma (LGG) (s_dis_ = − 3.26; *p* = 0.0; s_rand_ = − 6.1e−5; characteristic protein profiles increased in toLGG: p70S6K_pT389; increased in ToPRAD: YAP_pS127, HER2_pY1248, HER2, EGFR_pY1068, EGFR_pY1173, Src_pY416, and Cyclin_D1). Also, breast cancer cases which are similar to gastric cancer can be discriminated from cases that are similar to thyroid carcinoma with a discrimination score of s_dis_ = − 2.01 (*p* = 9.1e−5; s_rand_ = 3.0e−4). For breast cancer cases, similar to gastric cancer, characteristic proteins are ASNS and Cyclin_B1, whereas for cases similar to thyroid carcinoma, characteristic proteins with increased expression are Caveolin-1, Collagen_VI, PR, MAPK_pT202_Y204, and ER-alpha. Classes toSTAD and toTHCA can also be separated for gastric cancer with proteins characteristic to class toStad being Cyclin_B1, Caspase-7_cleavedD198, and Claudin-7 whereas toTHCA cases have significantly increased expression/phosphorylation of NF-kB-p65_pS536 and Caveolin-1.

A cross-cancer effect is present for the histotypes breast cancer and gastric cancer as classes toSTAD and toTHCA show overlapping characteristic proteins Cyclin_B1 and Caveolin-1 (*p* = 0.02) for those two histotypes. No other cross-cancer effects can be found.

In addition to the three previously published methods, we evaluated a classification which is based on a nonlinear principal component analysis of mutational profiles which yielded the genes TP53, TTN, and BRAF as indicators of high-level molecular types (Table 1). While it is obvious that these genes are not sufficient to establish a comprehensive molecular classification (particularly with respect to the controversial gene TTN [19–21]), they may be regarded as a very basic molecular typing system. This classification scheme assigns most cases to class 0 (48% of all cases). Classes 2, 4, and 6 (no BRAF mutation) are also very well populated from cases of different histological tumor types (13%, 20%, 11%). Overall, a molecular class on average includes cases from 10.75 histotypes (min 0; max 26). Cases of one histotype are assigned to 2.9 different classes on average (min 1; max 6).

The evaluation of protein profiles of the same histotype assigned to different classes yields an overall classification effectivity score of 16.8% which is almost as high as the score of the Ciriello classification despite its substantially lower complexity. The highest CES is found for adrenocortical carcinoma with 100%, although in this case only discriminable classes 0 and 4 have sufficient cases to be analyzed. Classes 0, 2, 4, and 6 are comprised of breast cancer cases, and their protein profiles are evaluated pairwise resulting in six comparisons. Of those six comparisons, only protein profiles of class 4 and 6 do not differ significantly. Therefore, the second highest classification effectivity score is found for breast cancer with 83.3% (five of six class to class comparisons). The class with the highest classification effectivity score is class 0 (no mutation in TP53, TTB, or BRAF) with 25.4%.

**Table 1:** Genetic complexity reduction-based classification scheme. The table is a complete illustration of the rules governing which class a case is assigned by the genetic complexity reduction-based classification

TP53 mutated	TTN mutated	BRAF mutated	Class
No	No	No	0
No	No	Yes	1
No	Yes	No	2
No	Yes	Yes	3
Yes	No	No	4
Yes	No	Yes	5
Yes	Yes	No	6
Yes	Yes	Yes	7

The highest class discriminability score for this classification is found in discriminating thyroid carcinoma protein profiles for classes 0 and 1 (s_dis_ = − 2.07; *p* = 0.0; s_rand_ = − 1.3e−4; characteristic protein profiles: ER-alpha, PR, GATA3, Bcl-2, and INPP4B increased for class 0—increased for class 4: Cyclin_B1 and ASNS). For breast cancer, differences in protein profiles are found between classes 0 and 6 (s_dis_ = − 1.85; *p* = 0.0; s_rand_ = 1.8e−5; characteristic protein profiles increased in class 0: ER-alpha, PR, GATA3, Bcl-2, Caveolin-1, AR, and INPP4B—for class 6: ASNS, Caspase-7_cleavedD198, and Cyclin_B1) and between class 0 and class 4 (s_dis_ = − 1.64; *p* = 0.0; s_rand_ = − 7.6e−5; characteristic proteins increased in class 0: ER-alpha, PR, GATA3, Bcl-2, and INPP4B—increased in class 4: Cyclin_B1 and ASNS).

For this classification, a consistent cross-cancer effect is found for classes 2 and 4 in both breast cancer and endometrial carcinoma. Protein profiles of classes 2 and 4 in breast cancer differ significantly for proteins ER-alpha, GATA3, AR, and ER-alpha_pS118 (increased in class 2), and Cyclin_B1 and p53 (increased in class 4). For endometrial carcinoma, protein profiles between class 2 and class 4 differ for ER-alpha, Akt_pS473, Akt_pT308, E-Cadherin, ER-alpha_pS118, Claudin-7, and CD49b (increased in class 2), and p53, Cyclin_B1, Cyclin_E1, and IGFBP2 (increased in class 4). With an overlap of four characteristic proteins (ER-alpha, ER-alpha_pS118, Cyclin_B1, and p53) this constitutes a significant (*p* = 0.0054) cross-cancer effect.

### Actionable genes are related to distinct protein profiles across cancers

While there are attempts to re-classify cancer based on comprehensive molecular profiles as outlined above, these are still largely theoretical considerations. Many so-called basket trials have already begun to evaluate the utility of *single* actionable mutations for targeted treatment selection independent of the histological tumor type. However, while some studies show that targeted therapies work in different histological tumor types (Anti-Her2 treatment in breast and gastric cancer, NTRK in various cancers [22]), several recent basket trials have demonstrated that the efficacy of targeted treatments often depends on the histological tumor type [13, 14]. Using the same approach as above, we are systematically evaluating all major actionable somatic mutations and copy number alterations against which drugs are approved for clinical use or which are currently tested in clinical trials with respect to their effects on proteins across cancers. Because we evaluate all actionable genes across all histotypes with sufficient numbers of mutated and wildtype cases, we both validate our approach by showing that established actionable genes show protein profile effects and identify novel gene-cancer combinations for which druggability is not yet established but for which we observe specific protein patterns. This integration of genomic and proteomic data allows us to predict genetic aberrations that are promising candidates for successful basket trials particularly if protein profile effects show a consistent directionality across cancers.

In our analysis, we study genes currently listed as actionable in the database OncoKB (http://oncokb.org/#/actionableGenes) that contains information on 476 genes in 65 histological tumor types and 97 related drugs [23] and that are sufficiently represented in The Cancer Genome Atlas (TCGA/TCPA). OncoKB lists 12 genes with level 1 evidence (FDA-approved), 11 genes with level 2 evidence (standard care), 26 genes in level 3 (clinical evidence), and 20 genes with biological evidence (level 4). We used all actionable genes from evidence levels 1 to 3 with simple somatic mutations (SNPs, insertions, deletions) and copy number variations with a sufficient number of mutated cases (12 actionable genes). A detailed list of evaluated actionable mutations can be found in Additional file 4: Table S3. We also analyzed actionable fusions using fusion data from [24], but due to the overall small number of fusions, there were only four tumor type fusion combinations that were analyzed. Results are given in Additional file 1, section “Actionable Fusions.”

Overall, our analysis showed for all analyzed 12 actionable genes (OncoKB evidence levels 1–3) that the mutational status is associated with significant differences in protein profiles in histotypes for which the respective targeted drugs are approved or currently being clinically tested and showed additional mutation-associated protein profiles in 9 histological tumor types. Moreover, our analysis identifies consistent cross-cancer effects for 4 genes (FGFR1, ERRB2, IDH1, KRAS/NRAS) in 11 histological tumor types. Only KRAS/NRAS mutations in colorectal cancer do not result in discriminable protein profiles comparing wild-type and mutated cases, whereas an effect can be observed for thyroid cancer and melanoma. To provide further evidence for the significance of this observation, we tested if protein profile discriminability is more often seen in tumor types where the gene is actionable than in non-actionable tumor types. This showed that the actionability of mutations is highly significantly associated with discriminable protein profiles (Fisher’s exact test *p* < 0.0001).

To evaluate if the protein profile analysis approach may contribute to predicting druggability of oncogenic mutations across cancers, we validated our predictions made for tumors from TCGA with drug response data available for cell lines. Our results demonstrate that in addition to confirming known druggable genes in the available cell line data, protein profile discriminability in between presence or absence of oncogenic mutations is predictive of drug response in cell line data across cancers (*p* = 0.048, Table 2). For more details, please see Additional file 1 section “Cell line analysis” and Additional file 5: Tables S4 and S5.

**Table 2:** Comparison between the results of cell line sensitivity of cell lines with actionable mutations and TCGA protein profile discriminability. For each actionable gene-histotype combination, Y = yes and N = no indicate whether cell lines with actionable mutations show different drug response than cell lines without actionable mutation and if protein profiles of actionable TCGA cases of the specific histotypes have been discriminable from non-actionable ones

Actionable gene	Histotype	Drug response	Differences in TCGA protein profiles
BRAF	SKCM*	**Y**	**Y**
ERBB2	BRCA*	**Y**	**Y**
	STAD*	**Y**	**Y**
	LUAD	**Y**	**Y**
	PAAD	**N**	**N**
EGFR	LUAD*	**Y**	**Y**
FGFR1	LUSC*	**Y**	**Y**
	COAD	**N**	**N**
	UCEC	N	Y
	SKCM*	**Y**	**Y**
KRAS	LUAD	**Y**	**Y**
	UCEC	N	Y
	COAD*	**N**	**N**
	STAD	**N**	**N**
	LUAD	**Y**	**Y**
MET	GBM	N	Y
	SKCM	**N**	**N**
	STAD	Y	N
	BRCA	N	Y
	KIRP	**Y**	**Y**
	KIRC*	**N**	**N**
	OV	N	Y
	LUAD	**Y**	**Y**
PIK3CA	BLCA	Y	N
	HNSC	**N**	**N**
	STAD	N	Y

*Histotypes where the gene is clinically established as actionable are marked. Fisher’s exact test shows there is a significant relation between protein discriminability and drug response (*p* = 0.0484)

bold: Response to targeted drugs and protein profile differences were consistently both present (Y) or both absent (N)

BRAF mutations are actionable in melanomas (OncoKB level 1). Mutations of BRAF are frequent enough in our data for melanoma (46% cases with mutation) and thyroid carcinoma (not yet reported by OncoKB, 56% cases with mutation) for further analysis. The actionable mutations create discriminable groups of cases for thyroid carcinoma (s_dis_ = − 2.07; *p* = 0.0; s_rand_ = − 1.0e−4) and melanoma (s_dis_ = − 0.10; *p* = 4.7e−3; s_rand_ = − 1.1e−4). For thyroid carcinoma, the protein expression of Fibronectin is altered. For melanoma, the levels of MAPK_pT202_Y204, PTEN, and Bcl-2 are decreased, and IGFBP2, E-Cadherin, Akt_pT308, and Akt_pS473 are increased. With no significant proteins intersecting between the two tumor types, no cross-cancer effect can be identified for BRAF.

CDK4 amplification is actionable for differentiated sarcomas (OncoKB level 2). CDK4 status testing reveals discriminable protein profiles for sarcomas and also for histotypes renal clear cell carcinoma, lung adenocarcinoma, breast cancer, ovarian carcinoma, thyroid carcinoma, low-grade glioma, adrenocortical carcinoma, and lung squamous cell carcinoma (all not yet reported by OncoKB). For sarcoma, 36% of the cases show CDK4 amplification and protein profiles are discriminable (s_dis_ = − 0.34; *p* = 0.0; s_rand_ = − 6.0e−5) with E-Cadherin, Caveolin-1, Akt_pS473, Cyclin_B1, ER-alpha, Akt_pT308, YAP_pS127, S6_pS240_S244, and Cyclin_E1 decreased and HSP70, Syk, Lck, Src_pY416, and Src_pY527 increased in CDK4 amplified cases. There is no cross similarity for CDK4.

EGFR mutations are actionable in non-small cell lung cancer (OncoKB level 1). Lung adenocarcinoma is the only histological tumor type with enough cases having an actionable mutation to perform our analysis. Lung adenocarcinoma (LUAD) cases with actionable mutation of EGFR are discriminable from those without by protein profile (s_dis_ = − 0.44; *p* = 5.9e−4; s_rand_ = 3.1e−5). EGFR_pY1068 levels are increased for cases with the respective mutations, and Claudin-7 levels are decreased among those cases.

**Fig. 5:**
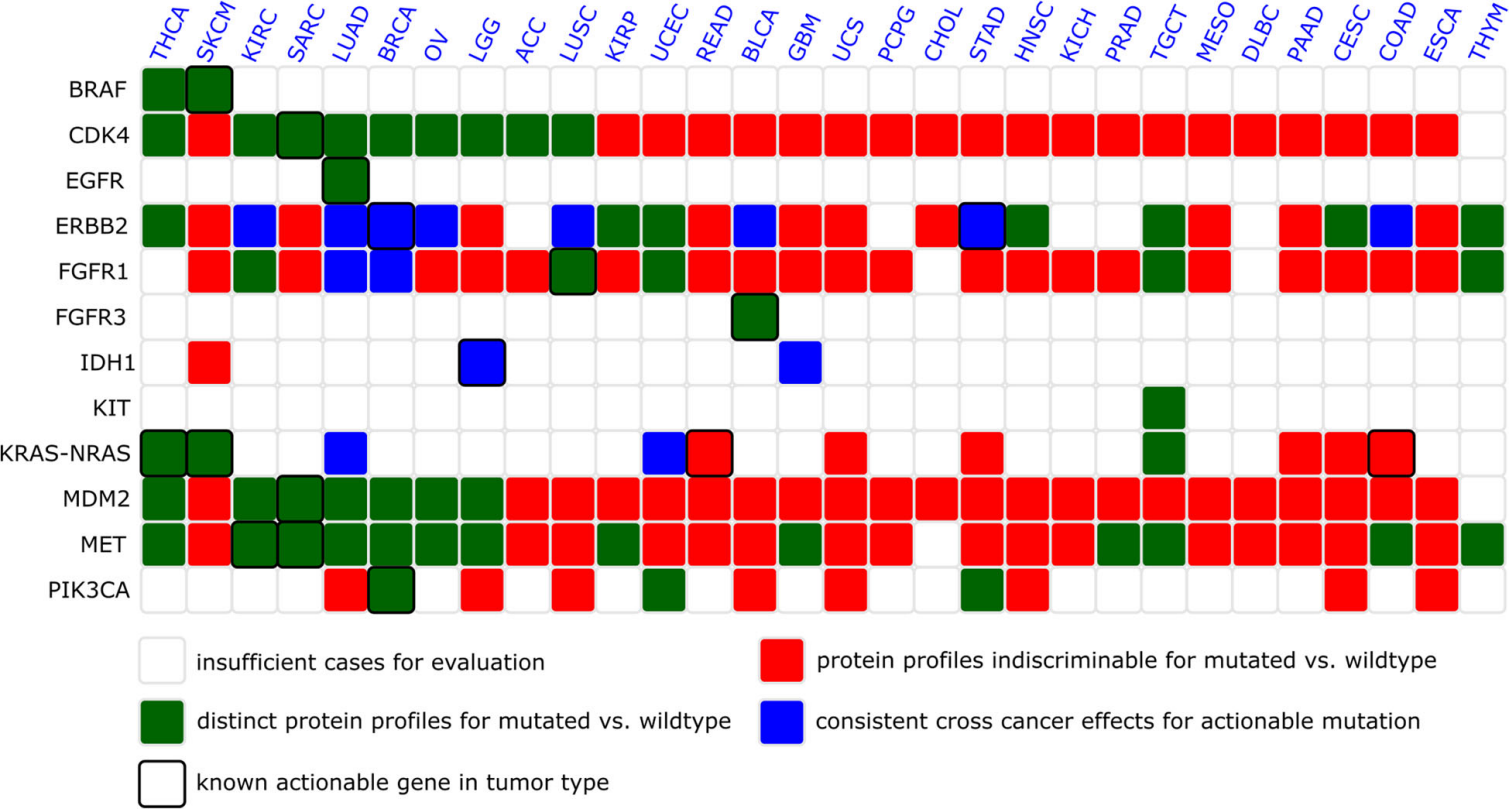
Analysis of differential effects of actionable mutations on protein profiles across cancers. Protein profiles are compared for cases with and without the respective actionable mutations. For certain cases, the presence of an actionable mutation has no visible effect on the level or proteins (red), for other cases (green) protein profiles are distinguishable for mutated vs. wild-type cases. Among these, pairs of four actionable genes and 11 cancer types exist, where the actionable mutations have a similar/same directional effect on protein profiles across cancers (blue) suggesting a similar (patho)mechanistic effect of the actionable mutations in these histological tumor types (compare also Fig. 6)

**Fig. 6:**
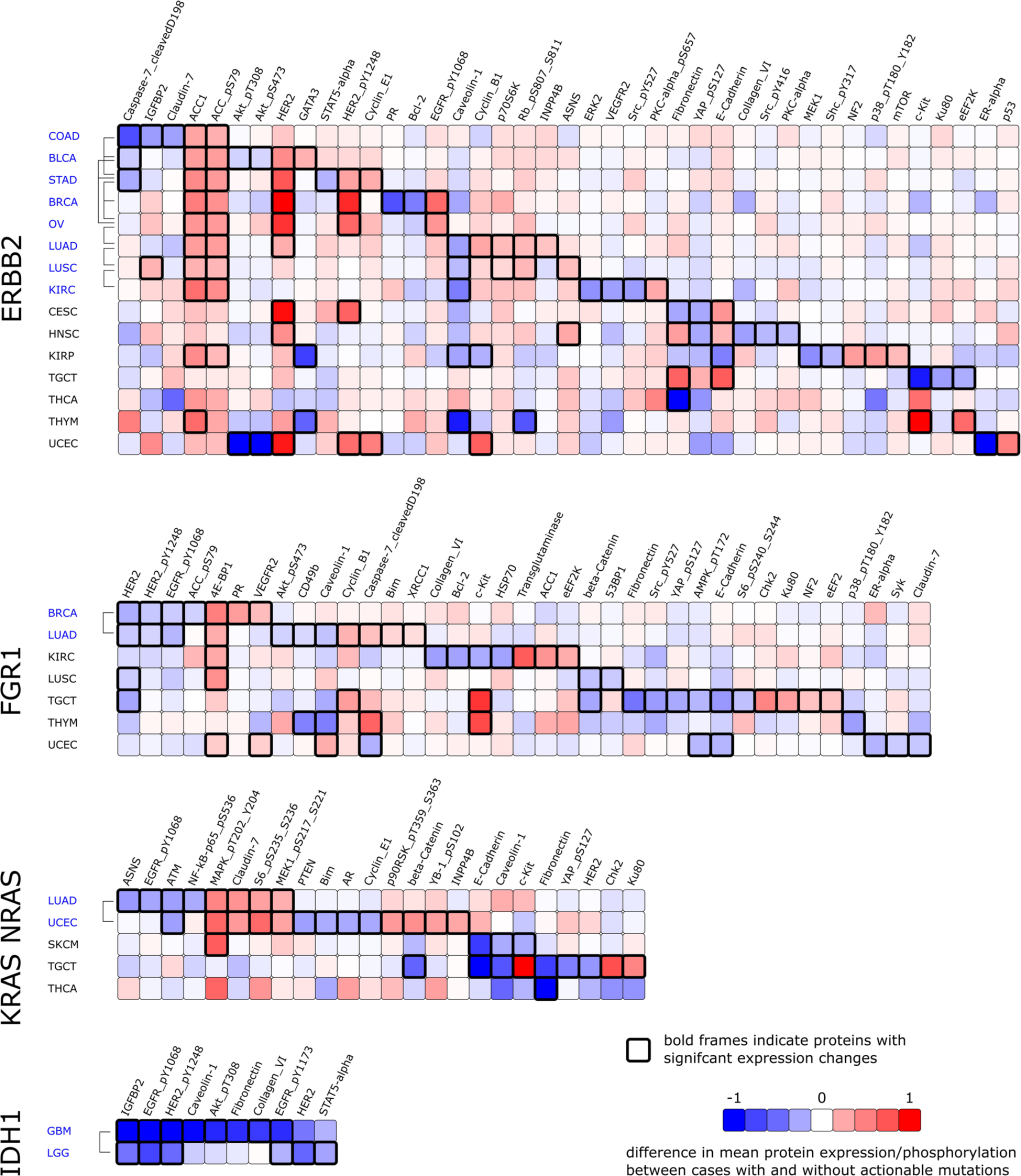
Identification of proteins characteristic of cross-cancer effects. Four out of the 12 studied actionable genes were found to have cross-cancer effects on the level of available protein profiles (compare Fig. 5). Proteins were evaluated for their role in discriminating between wild-type and mutated actionable genes. Bold border outlines indicate statistically significantly characteristic proteins for the given histological tumor type (rows). Brackets indicate the pairs of histological tumor types for which the actionable mutations show the same directional effects (indicated by an overlap of characteristic proteins with same directional change). Histotype names with cross-cancer effect are colored blue. For a comprehensive analysis of characteristic proteins for all actionable genes, see Additional file 1: Figure S5

ERBB2/HER2 amplification is actionable in breast cancer and gastric cancer (level 1 evidence, FDA-approved). We find that ERBB2/HER amplification status significantly influences protein expression profiles not only in breast and gastric cancer but also in lung squamous cell carcinoma and lung adenocarcinoma (in conformity with OncoKB level 4 data) and other 11 not yet reported histological types (endometrial carcinoma, renal papillary cell carcinoma, testicular germ cell tumors, urothelial bladder carcinoma, renal clear cell carcinoma, colon carcinoma, ovarian carcinoma, thymoma, thyroid carcinoma, cervical carcinoma, head and neck squamous cell carcinoma)—see also Fig. 5 or for details Additional file 3. For ten histological tumor types (melanoma, esophageal carcinoma, glioblastoma, mesothelioma, cholangiocarcinoma, rectal adenocarcinoma, sarcoma, pancreatic adenocarcinoma, uterine carcinosarcoma, low-grade glioma), no influence on available protein expression profiles is found. The discriminability score s_dis_ for Her2-amplified breast cancers is s_dis_ = − 0.43 (*p* = 0.0). The mean random discriminability score (mean discriminability score for one million random class assignments) is s_rand_ = − 6.2e−5. We identify characteristic proteins for ERBB2 amplification: HER2_pY1248, HER2, ACC1, and EGFR_pY1068 (increased levels in amplified cases) and Bcl-2 and PR (decreased levels—Fig. 6 illustrates in details characteristic proteins for ERBB2, FGFR1, IDH1, and KRAS-NRAS). For gastric cancer, actionable cases are discriminable from non-amplified cases (s_dis_ = − 0.18; *p* = 6.0e−6; s_rand_ = − 5.4e−5). The amplified cases show a decrease of Caspase-7_cleavedD198 and STAT5-alpha expression levels and an increase of HER2, ACC1, ACC_pS79, HER2_pY1248, and Cyclin_E1 expression levels. For ten pairs of histological tumor types, a similar change in protein expression —a cross-cancer effect— is found. Two histological tumor types in which ERBB2 amplification has a similar impact on proteins are breast (BRCA) and gastric (STAD) cancers (*p* = 2.3e−3). HER2_pY1248, HER2, and ACC1 have significantly higher mean levels in ERBB2-amplified cases in both histotypes. Other pairs of similarly influenced histological tumor types are lung adenocarcinoma and lung squamous cell carcinoma (*p* = 5.9e−6; proteins: Caveolin1, p70S6K, ACC_pS79, ACC1, Rb_pS807_S811) and breast cancer and ovarian carcinoma (*p* = 9.0e−6; proteins: HER2_pY1248, HER2, ACC1, EGFR_pY1068). For lung adenocarcinoma, cell line drug response data analysis shows sensitivity for cell lines with actionable mutation. This fact correlates to the cross-cancer effect of lung adenocarcinoma to breast cancer and gastric cancer, for which ERBB2 is actionable.

For FGFR1 amplification, clinical evidence (OncoKB level 3) exists on its actionability in lung squamous cell carcinomas. Our analysis shows that besides lung squamous cell carcinoma, protein expression of amplified cases is discriminable from non-amplified cases in renal clear cell carcinoma, testicular germ cell tumors, lung adenocarcinoma, endometrial carcinoma, breast cancer, and thymoma (all currently not reported by OncoKB). For lung squamous cell carcinoma, the protein profiles of the amplified cases have significant differences from those without amplification (s_dis_ = − 0.08; *p* = 4.1e−4; s_rand_ = 1.4e−5). Beta-Catenin, 53BP1, and HER2 are decreased in amplified cases whereas 4E-BP1 expression values are increased. A cross-cancer effect is found between breast cancer and lung adenocarcinoma with HER2, HER2_pY1248, and EGFR_pY1068 levels decrease and 4E-BP1 levels increase associated with FGFR1 amplification for both histological tumor types.

Certain FGFR3 mutations are actionable in bladder cancer (OncoKB level 3). Targetable FGFR3 mutations are only frequent enough in urothelial and bladder carcinoma for our analysis. The protein profiles of cases with at least one of these mutations are discriminable from the profiles of those without (s_dis_ = − 0.76; *p* = 2.7e−3; s_rand_ = − 1.3e−5). E-Cadherin, beta-Catenin, HER2, Ku80, PTEN, IRS1, and 53BP1 are increased among cases having one or more specific FGF3 mutation.

IDH1 mutations are actionable in acute myeloid leukemia, cholangiocarcinoma, and glioma (OncoKB level 3). Specific IDH1 mutations lead to discriminable protein profiles for low-grade glioma (s_dis_ = − 0.47; *p* = 0.0; s_rand_ = − 3.1e−6) and glioblastoma (s_dis_ = − 1.59; *p* = 5.0e−4; s_rand_ = − 1.0e−5). For low-grade glioma cases with an actionable mutation, protein profiles of EGFR_pY1068, HER2_pY1248, HER2, IGFBP2, EGFR_pY1173, and STAT5-alpha are decreased. For glioblastoma IGFBP2, EGFR_pY1068, HER2_pY1248, Caveolin-1, Akt_pT308, Fibronectin, Collagen_VI, and EGFR_pY1173 are decreased in the group of mutated cases. Therefore, IGFBP2, EGFR_pY1068, HER2_pY1248, and EGFR_pY1173 are affected in the same way by IDH1 mutations in low-grade glioma and glioblastoma, and we report a cross-cancer effect for those groups.

KIT mutations are actionable in gastrointestinal stromal tumors (OncoKB level 1). For the tested KIT mutations, only testicular germ cell tumors (TGCT) had enough mutated cases sufficient for our analysis. The protein profiles of the mutated and wild-type cases are discriminable (s_dis_ = − 0.90; *p* = 4.5e−3; s_rand_ = − 2.6e−4) with decreased E-Cadherin and Fibronectin expression in wildtype cases and increased c-Kit, STAT5-alpha, and Syk expression levels. The results for KIT presented above are those for mutations typically treated with Imatinib; we also tested mutations treated with other KIT-inhibitory drugs yielding similar results.

KRAS/NRAS mutations are therapeutically relevant for melanomas, colorectal cancer, and thyroid cancer (OncoKB level 3). Specific KRAS/NRAS mutations are correlated with differences in protein profiles for melanomas and thyroid cancer and also for testicular germ cell tumors, endometrial carcinoma, and lung adenocarcinoma (in conformity with OncoKB level 4 data). No discriminability of protein profiles can be found for colon carcinoma (s_dis_ = − 5.3e−3; *p* = 0.43; s_rand_ = − 6.2e−3) and rectum adenocarcinoma (s_dis_ = + 0.16; *p* = 0.59; s_rand_ = 0.027). KRAS/NRAS mutations in colorectal adenomas are the only occurrence where we cannot find discriminable protein profiles between cases that would be treated and those which would not. For melanoma (s_dis_ = − 0.16; *p* = 1.0e−3; _and_ = 5.2e−6) E-Cadherin, Caveolin-1, and c-Kit expression levels are decreased for mutated cases, and MAPK_pT202_Y204 is increased. For thyroid carcinoma (s_dis_ = − 1.53; *p* = 0.0; s_rand_ = − 3.0e−4), the level of Fibronectin is decreased in mutated cases. For lung adenocarcinoma and endometrial carcinoma, we observed a cross-cancer effect for KRAS/NRAS-mutated cases as ATM levels are decreased, and MAPK_pT202_Y204, Claudin-7, S6_pS235_S236, and MEK1_pS217_S221 are increased in both histological tumor types consistently.

MDM2 amplification is actionable in liposarcoma (OncoKB level 3). Besides sarcoma, the protein profiles of cases with MDM2 amplification are discriminable from those with normal copy numbers for renal clear cell carcinoma, lung adenocarcinoma, thyroid carcinoma, breast cancer, ovarian carcinoma, and low-grade glioma (all currently not reported by OncoKB). Protein levels of sarcoma cases with MDM2 amplifications are discriminable from those without, with a dissimilarity score of s_dis_ = − 0.41 (*p* = 0.0; s_rand_ = − 8.9e−5). Amplified cases show decreased levels of E-Cadherin, Akt_pS473, Akt_pT308, ER-alpha, Caveolin-1, S6_pS240_S244, S6_pS235_S236, and Cyclin_B1 and increased levels of HSP70, Syk, and Lck. No consistent cross-cancer effect is found.

MET amplification is actionable in non-small cell lung cancers and renal cell carcinoma (OncoKB level 2). In addition to these histotypes, we found 11 other histological tumor types (renal clear cell carcinoma, low-grade glioma, renal papillary cell carcinoma, colon carcinoma, thyroid carcinoma, thymoma, sarcoma, lung adenocarcinoma, testicular germ cell tumors, prostate adenocarcinoma, glioblastoma, breast and ovarian carcinoma) where MET amplification is associated with a significant change in protein expression. For lung adenocarcinoma, the discriminability score is s_dis_ = − 0.067 (*p* = 1.5e−3; s_rand_ = 2.0e−5). Proteins that are characteristic of MET amplification status are cyclin_E1, ASNS, cyclin_B1, ACC1, Fibronectin, and ACC_pS79 (increased levels), and c-Kit, Caveolin-1, and Claudin-7 (decreased levels). MET amplification is present in renal clear cell carcinoma cases, and protein profiles of amplified and non-amplified cases can be discriminated (s_dis_ = − 0.18; *p* = 0.0; s_rand_ = − 2.0e−5; Src_pY527, Bcl-2, beta-Catenin, PTEN, MAPK_pT202_Y204 are decreased in amplified cases and ACC1, Cyclin_B1, ASNS, ACC_pS79, and Transglutaminase are increased). We did not observe any similar effect of MET amplification on protein expression in other histological tumor types.

PIK3CA activating mutations are actionable for breast cancer (OncoKB evidence level 3). Besides breast cancer, we find an impact on protein profiles for gastric cancer and endometrial carcinoma. OncoKB level 4 data lists all available histological tumor types as possibly actionable for PIK3CA activating mutations. Yet no protein level effect is found for cervical carcinoma, head and neck squamous cell carcinoma, lung adenocarcinoma, urothelial bladder carcinoma, esophageal carcinoma, low-grade glioma, lung squamous cell carcinoma, and uterine carcinosarcoma. Breast cancer cases with PIK3CA-activating mutations are discriminable from those without (s_dis_ = − 0.52; *p* = 0.0; s_rand_ = 6.2e−6) with specific proteins (increased levels) PR, ER-alpha, MAPK_pT202_Y204, Fibronectin, AR, and GATA3 in mutated cases and Cyclin_B1, Cyclin_E1, ASNS, and HER2 being decreased. As there is no significant overlap of altered proteins between breast cancer, gastric cancer, and endometrial carcinoma, no cross-cancer effect was found.

## Discussion

Although clinical parameters still outweigh the relevance of molecular profiles for predicting patient survival [25], genomic medicine predicting therapies driven by next-generation sequencing techniques has started to transform diagnostics and oncological therapy during the last decade from a discipline that largely relied on conventional chemotherapies to one that increasingly exploits knowledge on therapeutically targetable oncogenic mechanisms. The view on molecular properties has questioned the relevance of organ- and tissue-typing in tumors and led to proposals to focus on molecular rather than histological concepts of cancer classification. However, many open questions remain because apart from mutations with unknown functional effects, it is often not possible even for oncogenic mutations with established clinical relevance in one cancer type to transfer knowledge of actionability to another cancer type. Moreover, even for a given cancer, clinical response to therapy targeting a specific mutation strongly varies, which is likely due to the modulatory influence of the usually high mutational complexity in tumors.

Because proteins carry out most genetically defined cellular functions, our computational analysis relates genetic alterations and histology with protein profiles to estimate their functional effects and offer a way of evaluating molecular tumor classifications and actionable genes across cancers. To this end, we measured how classes from the four studied molecular classifications are discriminable on the level of protein expression and phosphorylation based on a panel of 120 cancer-associated proteins available through The Cancer Proteome Atlas. The results showed only a partial histotype-independence, which is indicated by overall low classification effectivity scores for those classification that rely only on mutational profiles (Ciriello CES = 17.5%, Heim CES = 2%, genetic complexity reduction-based classification CES = 16.8%). In contrast to this, the classification by Hoadley et al. which combines genomic and proteomic information shows CES of 80% indicating that proteins have a substantial influence. Our results also show that even though a number of molecular classes of these four molecular classifications are reflected by the protein profiles, the characteristic proteins of the different classes are not identical. This indicates that identical genetic alterations are not translated into protein profiles in the same way in different histotypes. The only exception was one cross-cancer effect found in the classification by Heim et al. where the protein profiles between classes “toSTAD” and “toTHCA” showed same directional changes in breast cancer and gastric cancer (of note, more cross-cancer effects were found for the actionable gene analysis, see below).

These observations are largely consistent with the results of the actionable gene analysis which showed specific protein signatures for 12 actionable genes in the corresponding cancer types from the OncoKB database. In addition to showing that our analysis identifies protein-level effects for known actionable genes and corresponding cancer types, our approach also identified protein-level alterations indicative of potential novel actionable gene—cancer combinations that are so far unknown according to OncoKB including level 4 evidence (biological information). This includes ERBB2/HER2 amplification in endometrial carcinoma, renal papillary carcinoma, testicular germ cell tumors, urothelial carcinoma, renal clear cell carcinoma, colon carcinoma, ovarian carcinoma, thymoma, thyroid carcinoma, cervical carcinoma, and head and neck squamous cell carcinoma. For MET amplification, our approach predicts effects for renal clear cell carcinoma, low-grade glioma, renal papillary carcinoma, colon carcinoma, thyroid carcinoma, thymoma, sarcoma, lung adenocarcinoma, testicular germ cell tumors, prostate adenocarcinoma, glioblastoma, breast cancer, and ovarian carcinoma; for FGFR1 amplification: renal clear cell carcinoma, testicular germ cell tumors, lung adenocarcinoma, endometrial carcinoma, breast cancer, thymoma; MDM2 amplification renal clear cell carcinoma, lung adenocarcinoma, thyroid carcinoma, breast cancer, ovarian carcinoma, and low-grade glioma; BRAF V600: thyroid carcinoma; CDK4 amplification: renal clear cell carcinoma, lung adenocarcinoma, breast cancer, ovarian carcinoma, thyroid carcinoma, low-grade glioma, adrenocortical carcinoma, and lung squamous cell carcinoma.

Among the predicted genes, the most promising candidates for cross-cancer therapies are ERBB2/Her2, FGFR1, IDH1, and KRAS because our analysis finds consistent protein profile changes across histotypes (ERBB2/Her2 among others breast cancer – gastric cancer, FGFR1: breast cancer – lung adenocarcinoma, IDH1: low-grade glioma – glioblastoma and KRAS: lung adenocarcinoma – endometrial carcinoma) for these genes. The fact that our approach also identifies the well-known trans-cancer efficacy of ERBB2/Her2 inhibition in breast and gastric cancer [7, 8] supports the potential clinical value of our predictions.

Interestingly, actionable genes with copy number alterations showed effects on protein expression for more histotypes than those with simple somatic mutations (10.2 affected tumor types on average for amplifications vs. 2.14 for simple somatic mutations).

To validate our findings, we used drug response data from cell line repositories. The results show that our computational proteomic analysis using data from The Cancer Genome Atlas correctly predicts drug response in an independent data set of cell lines.

A study also linking proteomic and mutation data by Akbani et al. [26] clusters cases based on proteomic data, but clustering is evaluated by survival time. Akbani et al. also report relations between differences in frequencies of certain mutations between clusters and the differences in survival statistics for these clusters. In contrast to this study, our approach relies on mutational data as clustering or classification input and evaluates classification based on proteomic data and is therefore also capable of evaluating the effect of a single mutation directly.

A limitation of our study is that data on only 120 proteins measured for all cases were available. It cannot be excluded (and is perhaps even likely) that more comprehensive protein profiles would lead to the identification of additional observable protein-level effects of genetic classifications because in the currently available profiles, certain aspects of cellular function are simply not covered. However, the proteins included in the panel had been selected to represent major cancer-related functional and signaling pathways such as, for example, DNA damage, hormone signaling, and proliferation deduced from a comprehensive mass spectrometry-based dataset [26]. While this may still lead to a slightly too pessimistic view on the global molecular tumor classifications, it is therefore unclear to what extent the inclusion of more proteins would add to functionally and clinically relevant information. With respect to the actionable gene analysis, our approach may underestimate the number of potentially druggable genes, but the fact that it readily identifies many well-established actionable gene, cancer combinations, such as, for instance, HER2 amplification in breast and gastric cancer [7, 8], indicates its validity.

The approach we present facilitates analyses of the relationship between genomic and proteomic profiles in the context of different histological tumor types. It is important to note that the ability of our study to reveal cross-cancer effects is limited by the molecular classifications we evaluate here. With the advance of our understanding of cancer, improved molecular classifications and more detailed definitions of the actionability of genes will become available. The method presented in this study is independent of those changes and therefore can be also applied to evaluate future definitions. Future molecular tumor typing concepts are likely to also include additional aspects such as intratumoral heterogeneity which will become increasingly important for the interpretation of molecular profiles [27, 28].

## Conclusions

While the current tumor classification system is still largely based on histology, it will be increasingly complemented by molecular profiling to meet the requirements of precision medicine. Our analysis shows that tumor typing solely based on mutational profiling is incomplete. By evaluating protein-level effects of genetic aberrations, our approach facilitates the identification of functionally relevant mutations and may therefore contribute to predicting actionable mutations across cancers and to guide basket trial design.

## Additional files

Additional file 1: Results for actionable fusions, details on genetic complexity reduction algorithm, method description of evaluating relations between mutational and protein profiles, method description of cell line analysis, an analysis on cross-cancer similarities for different gene sets, a figure for characteristic proteins on all actionable genes and information on batches not used due to possible batch effects. (PDF 1511 kb)

Additional file 2: Ciriello pathway analysis results. A table containing detailed results of the Ciriello pathway analysis. (XLSX 19 kb)

Additional file 3: Complete listing of results. Detailed listing of the results of discriminability and cross cancer effect analysis. (CSV 210 kb)

Additional file 4: Actionable genes. A table with details on all actionable genes analyzed. (XLSX 14 kb)

Additional file 5: Cell lines analysis results. Two tables containing results on cell line data analysis. (XLSX 14 kb)

## References

[1] Flaherty KT, Puzanov I, Kim KB, Ribas A, McArthur GA, Sosman JA (2010). Inhibition of mutated, activated BRAF in metastatic melanoma. N Engl J Med.

[2] Kris MG, Natale RB, Herbst RS, Lynch TJ, JR PD, Belani CP (2003). Efficacy of gefitinib, an inhibitor of the epidermal growth factor receptor tyrosine kinase, in symptomatic patients with non-small cell lung cancer: a randomized trial. JAMA.

[3] Perez EA, Romond EH, Suman VJ, Jeong J-H, Davidson NE, Geyer CE (2011). Four-year follow-up of trastuzumab plus adjuvant chemotherapy for operable human epidermal growth factor receptor 2-positive breast cancer: joint analysis of data from NCCTG N9831 and NSABP B-31. J Clin Oncol.

[4] Ciriello G, Miller ML, Aksoy BA, Senbabaoglu Y, Schultz N, Sander C (2013). Emerging landscape of oncogenic signatures across human cancers. Nat Genet.

[5] Hoadley KA, Yau C, Wolf DM, Cherniack AD, Tamborero D, Ng S (2014). Multiplatform analysis of 12 cancer types reveals molecular classification within and across tissues of origin. Cell.

[6] Weinstein JN, Collisson EA, Mills GB, Shaw KR, Ozenberger BA, Ellrott K (2013). The Cancer Genome Atlas pan-cancer analysis project. Nat Genet.

[7] Bang Y-J, van Cutsem E, Feyereislova A, Chung HC, Shen L, Sawaki A (2010). Trastuzumab in combination with chemotherapy versus chemotherapy alone for treatment of HER2-positive advanced gastric or gastro-oesophageal junction cancer (ToGA): a phase 3, open-label, randomised controlled trial. Lancet.

[8] Rugo HS, Barve A, Waller CF, Hernandez-Bronchud M, Herson J, Yuan J (2017). Effect of a proposed trastuzumab biosimilar compared with trastuzumab on overall response rate in patients with ERBB2 (HER2)-positive metastatic breast cancer: a randomized clinical trial. JAMA.

[9] Carvajal RD, Lawrence DP, Weber JS, Gajewski TF, Gonzalez R, Lutzky J (2015). Phase II study of nilotinib in melanoma harboring KIT alterations following progression to prior KIT inhibition. Clin Cancer Res.

[10] Macleod AC, Klug LR, Patterson J, Griffith DJ, Beadling C, Town A, Heinrich MC (2014). Combination therapy for KIT-mutant mast cells: targeting constitutive NFAT and KIT activity. Mol Cancer Ther.

[11] Souglakos J, Philips J, Wang R, Marwah S, Silver M, Tzardi M (2009). Prognostic and predictive value of common mutations for treatment response and survival in patients with metastatic colorectal cancer. Br J Cancer.

[12] Yokota T, Ura T, Shibata N, Takahari D, Shitara K, Nomura M (2011). BRAF mutation is a powerful prognostic factor in advanced and recurrent colorectal cancer. Br J Cancer.

[13] Hyman DM, Puzanov I, Subbiah V, Faris JE, Chau I, Blay J-Y (2015). Vemurafenib in multiple nonmelanoma cancers with BRAF V600 mutations. N Engl J Med.

[14] Lopez-Chavez A, Thomas A, Rajan A, Raffeld M, Morrow B, Kelly R (2015). Molecular profiling and targeted therapy for advanced thoracic malignancies: a biomarker-derived, multiarm, multihistology phase II basket trial. J Clin Oncol.

[15] Ramos AH, Lichtenstein L, Gupta M, Lawrence MS, Pugh TJ, Saksena G (2015). Oncotator: cancer variant annotation tool. Hum Mutat.

[16] Broad Institute TCGA Genome Data Analysis Center (2016). Analysis-ready standardized TCGA data from Broad GDAC Firehose stddata__2015_11_01 run: Broad Institute of MIT and Harvard.

[17] Li J, Lu Y, Akbani R, Ju Z, Roebuck PL, Liu W (2013). TCPA: a resource for cancer functional proteomics data. Nat Meth.

[18] Heim D, Budczies J, Stenzinger A, Treue D, Hufnagl P, Denkert C (2014). Cancer beyond organ and tissue specificity: next-generation-sequencing gene mutation data reveal complex genetic similarities across major cancers. Int J Cancer.

[19] Kim N, Hong Y, Kwon D, Yoon S (2013). Somatic mutaome profile in human cancer tissues. Genomics Inform.

[20] Kim Y-A, Madan S, Przytycka TM (2017). WeSME: uncovering mutual exclusivity of cancer drivers and beyond. Bioinformatics.

[21] Lawrence MS, Stojanov P, Polak P, Kryukov GV, Cibulskis K, Sivachenko A (2013). Mutational heterogeneity in cancer and the search for new cancer genes. Nature.

[22] Ricciuti B, Brambilla M, Metro G, Baglivo S, Matocci R, Pirro M, Chiari R (2017). Targeting NTRK fusion in non-small cell lung cancer: rationale and clinical evidence. Med Oncol.

[23] Chakravarty D, Gao J, Phillips S, Kundra R, Zhang H, Wang J, et al. OncoKB: a precision oncology knowledge base. JCO Precis Oncol. 2017:1–16. 10.1200/PO.17.00011. 10.1200/PO.17.00011 PMC5586540 28890946

[24] Hu X, Wang Q, Tang M, Barthel F, Amin S, Yoshihara K (2018). TumorFusions: an integrative resource for cancer-associated transcript fusions. Nucleic Acids Res.

[25] Yuan Y, van Allen EM, Omberg L, Wagle N, Amin-Mansour A, Sokolov A (2014). Assessing the clinical utility of cancer genomic and proteomic data across tumor types. Nat Biotechnol.

[26] Akbani R, Ng PK, Werner HM, Shahmoradgoli M, Zhang F, Ju Z (2014). A pan-cancer proteomic perspective on The Cancer Genome Atlas. Nat Commun.

[27] Morris LG, Riaz N, Desrichard A, Senbabaoglu Y, Hakimi AA, Makarov V (2016). Pan-cancer analysis of intratumor heterogeneity as a prognostic determinant of survival. Oncotarget.

[28] Andor N, Graham TA, Jansen M, Xia LC, Aktipis CA, Petritsch C (2016). Pan-cancer analysis of the extent and consequences of intratumor heterogeneity. Nat Med.

[29] Smirnov P, Safikhani Z, El-Hachem N, Wang D, She A, Olsen C (2016). PharmacoGx: an R package for analysis of large pharmacogenomic datasets. Bioinformatics.

