# Supplementary material for: Computational analysis reveals histotype-dependent molecular profile and actionable mutation effects across cancers
Source: Genome Med. 2018 Nov 15;10:83. doi: 10.1186/s13073-018-0591-9 (PMC6238410; doi:10.1186/s13073-018-0591-9)
Supplement: Supplementary file 1 — Results for actionable fusions, details on genetic complexity reduction algorithm, method description of evaluating relations between mutational and protein profiles, method description of cell line analysis, an analysis on cross-cancer similarities for different gene sets, a figure for characteristic proteins on all actionable genes and information on batches not used due to possible batch effects. (PDF 1511 kb) [file 13073_2018_591_MOESM1_ESM.pdf]

Supplementary Material

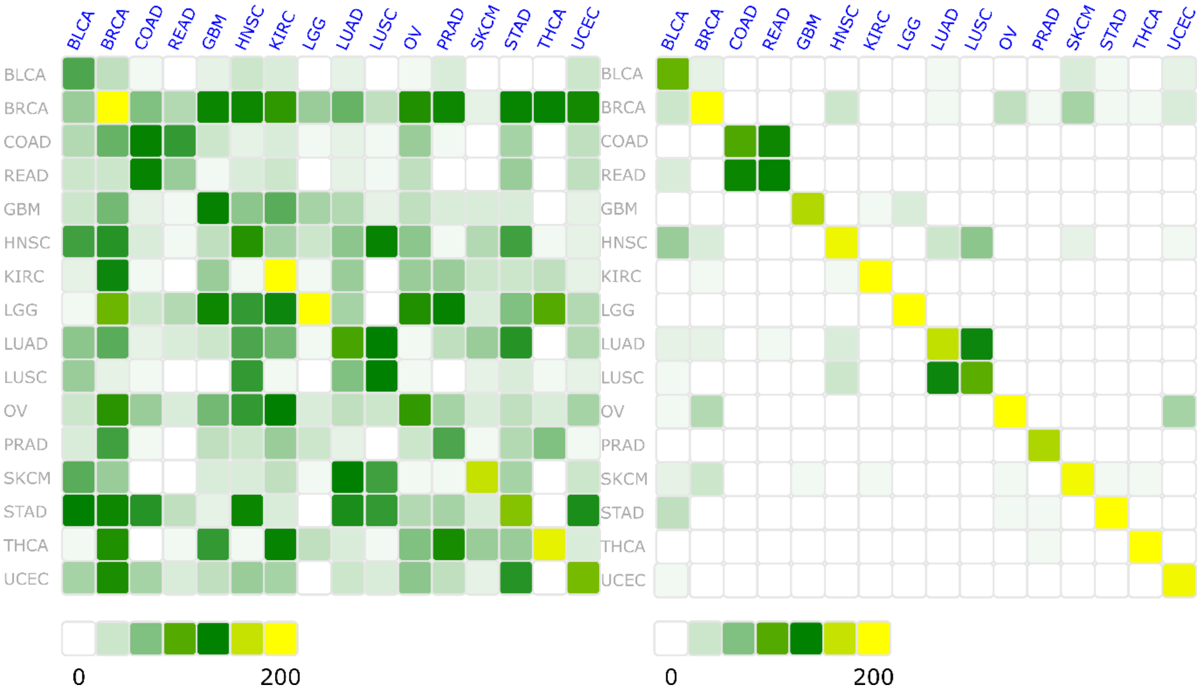

Figure S1: Heatmaps representation of results shown as chord diagrams in Figure 2 of the main manuscript. The number of cases of a histotype (row) with a most similar case from histotype (column) is shown. For the left heatmap the most similar case is calculated based on mutational overlap between to cases, for the right figure similarity is calculated by using TCPA protein expression values.

Actionable Fusions

For the nine analyzed targeted fusions only for three of them there were five or more cases with that fusion from the same tumor type. For BCR-ABL1, ALK, PDGFR, ROS1, BRAF, PCM1-JAK2 there were not enough cases of the same tumor type to run our analysis.

FGFR2/3 fusions are actionable in bladder cancer (OncoKB level 3). There were enough cases with FGFR2/3 fusion to be analysed for bladder cancer and lung squamous carcinoma. Bladder cancer cases with FGFR2/3 fusion are discriminable from those without by protein profile (s_{dis}=-1.4; p=8.9e-5; s_{rand}=1.8e-4). E-Cadherin, Src_pY527, Rb_pS807_S811, Src, GATA3, HER2, Claudin-7, INPP4B, p38_pT180_Y182, Src_pY416 and beta-Catenin levels are increased for cases with FGFR2/3 fusion Fibronectin, Caspase-7_cleavedD198, Caveolin-1, Transglutaminase levels are decreased among those cases. For lung squamous carcinoma protein profiles of cases with fusion were not discriminable from those without (s_{dis}=0.19; p=0.755; s_{rand}=0.89e-4).

NTRK fusions are actionable in all solid cancers (OncoKB level 3). Only for thyroid carcinoma there were enough cases with NTRK fusion to be analysed and cases were discriminable by protein profile (s_{dis}=-0.91; p=0.042; s_{rand}=0.49e-4).

RET fusions are actionable in non-small cell lung cancer (OncoKB level 3). Thyroid carcinoma is the only tumor type with enough cases with RET fusion to be analysed. Protein profiles of cases of that tumor type with RET fusion are discriminable from protein profiles of cases without (s_{dis}=-0.54; p=0.043; s_{rand}=0.19e-4).

Materials and Methods: Genetic complexity reduction

TP53, TTN, and BRAF are the genes that identify the coarse cluster structure of the data when mutational profiles are represented as a two-dimensional vectors. For the collection of mutational profiles x_1,...,x_N, their 2D representation is obtained by building the matrix K of size N x N with entries K_ij = log(epsilon + <x_i,x_j>)), and computing the first eigenvectors u_1 and u_2 of that matrix. The 2D representation of the ith mutational profile is then given by the value of the ith row of u_1 and u_2. The collection of mutational profiles can therefore be represented as a scatter plot (see supplementary Fig. 1). The clustering structure is stable under a large range of values of epsilon. In the plot, points are color-coded by the states of genes TP53, TTN, BRAF, where the binary triplets indicate their respective mutation.

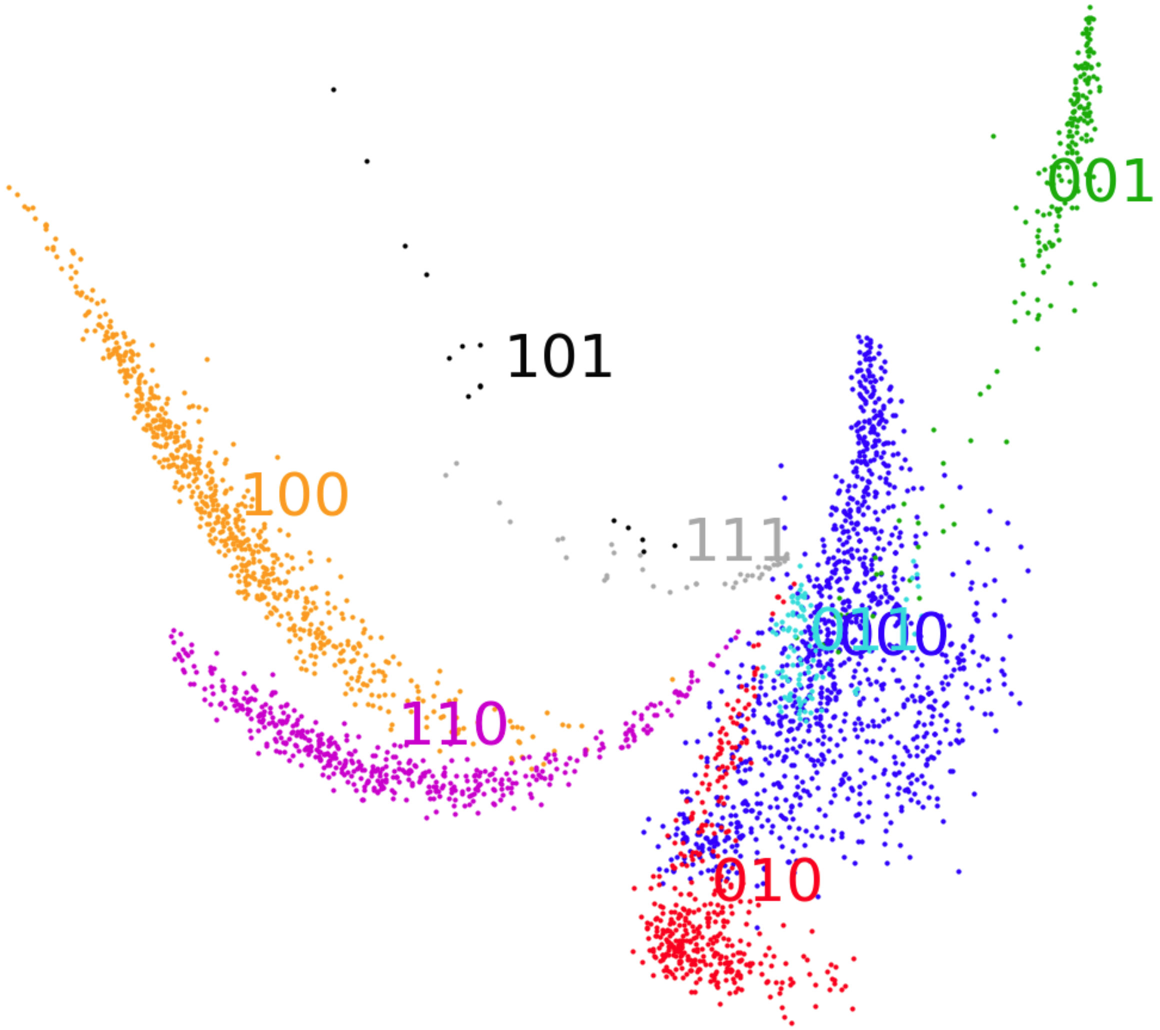

Figure S2: Genetic complexity reduction: 2d plot of principle component analysis of the mutational space. Each dot refers to the one case. Dots are colored by their class assigned by the genetic complexity reduction classification. A three-digit code is used to define the classes. Each digit refers to on gene where 0 is associated with wildtype and 1 with mutation. The first digit refers to TP53, the second to TTN and the third to BRAF. So, for example class 001 is comprised of cases without TP53 and TTN but with BRAF mutation.

Materials and Methods: The relation between mutational and protein profiles

To further examine the relation between mutational profiles and protein profiles and to identify potential convergences of different mutations (in the same pathway) into similar protein profiles, we performed a gene set enrichment analysis. For each pair of distinct Ciriello (mutational => M) classes, which show similar protein profiles by our approach, we searched for gene sets common to both classes that could explain the absence of protein profile discriminability. For our analysis we used GSEA C2: curated gene sets, which covers a broad range of processes. In addition to the standard enrichment analysis, we evaluated if the gene set contains a significant number of genes that correspond to (phospho-)proteins for which expression values are significantly de/increased.

The following steps were performed for each gene set/pathway and each pair of Ciriello groups: We first determined for each case if the number of mutated genes is increased within the pathway/gene set. We used Fisher's exact test to evaluate this (number of genes mutated within the pathway, number of genes mutated not in the pathway, number of genes of the pathway not mutated and not number of genes neither mutated nor in the pathway). We set the mutational criterion for enrichment of a gene set to be met when more than 33% of cases of both Ciriello groups had an increased overlap of mutated genes with genes of this gene set. We classified a pathway/gene set to be enriched in a pair of Ciriello groups if, in addition to this overlap of mutations, the proteins characteristic for the two groups overlapped with the genes of the gene set (also done by Fisher's exact test). (Phospho-) proteins were considered as characteristic of pairs of Ciriello groups if the difference between the mean expression value of the cases of the two groups and the mean expression value of the rest of the cases of the histotpye differed. The significance of this difference was evaluated in 10000 Monte Carlo Runs (comparing the difference to the mean expression value of this protein in this histotpye to the difference between the mean of randomly composed groups and the histotpye mean). By this procedure we found 0 to n gene sets enriched in (associated to) each pair of Ciriello groups.

We analysed a total of 76 class pairs. For 24 of those pairs, at least one gene set was identified as enriched and associated with similar protein profiles. For the other 52 pairs, no common gene sets potentially explaining the convergence of mutations into similar protein profiles was found. To identify relevant molecular similarities across different histotypes we searched for pairs of Ciriello classes which we found to be associated with gene sets across different tumor types. 16 Ciriello class pairs occurred in more than one tumor type. For eight of those 16 pairs a gene set was enriched in no tumor type. For six pairs, a gene set was associated with only one tumor type. For the group pair M5 - M6 gene sets were enriched for breast cancer cases (set: ST_ADRENERGIC, PID_ERBB1_RECEPTOR_PROXIMAL_PATHWAY) and uterine cancer cases (sets: KEGG_ENDOMETRIAL_CANCER, BIOCARTA_HCMV_PATHWAY, BIOCARTA_ERK5_PATHWAY etc.). However, there was no intersection between gene sets enriched in breast cancer and uterine cancer. For groups M4 and M6 in endometrial cancer 22 enriched gene sets were identified. For groups M4 - M6 in kidney renal cancer we found the gene set TCGA_GLIOBLASTOMA_MUTATED to be enriched. This gene set is also one of the gene sets enriched in this group pair for endometrial cancer. This is the only example in which the same gene set was enriched in a pair of Ciriello classes across different histotypes.

The analysis shows that when using gene sets, 24 (of 76) combinations of pairs of Ciriello classes and histotypes exist that have similar protein profiles which are associated with a common gene set (results in Additional file 2). However, for 52 out of 76 pairs of molecular classes the gene set enrichment did not provide any mutation-protein-relations.

Possible explanations include that an underlying functional reason exists but is not known yet (and thus not covered by the gene set data) or that the limited available protein data (RPPA <200 proteins) does not cover the relevant mechanisms. The fact that only for one pair of classes the same gene set was found enriched in two different tumor types, further supports one of the conclusions of our study that the functional implications of mutations are often histotype-dependent.

Materials and Methods: Cell line analysis

For an additional analysis relating protein profiles to response to targeted therapy, we use drug response data for cell line data available through PharmacoGx https://pharmacodb.pmgenomics.ca/pharmacogx) for 1488 cell lines, and MCLP for (phospho)-protein cell line data).
To evaluate if the protein profile analysis approach as presented in our manuscript may contribute to predicting druggability of oncogenic mutations across cancers, we validated our predictions made for tumors from TCGA with drug response data available for cell lines.

More precisely, we searched within the cell line drug response data for drugs included in our analysis of actionable genes from the OncoKB data base (http://oncokb.org). For each actionable gene and each cell line, we then evaluated somatic mutation and copy number alteration data to identify cell lines with and without the respective actionable genetic alterations. We then compared drug response (ic50) of cell lines with and without actionable mutations grouped by histotypes. To identify statistically significant differences in drug response values we used the Wilcoxon-Mann-Whitney-Test (U-Test) and conducted the analysis when at least 5 cell lines were available in the actionable and non-actionable group. Additionally, we searched for an over representation of IC50 values below 1μM (sensitive cases) within the actionable group using Fisher's exact test. For both tests we used a significance level of 0.1 because of our observations, that for many known and clinically established targeted therapies the difference between ic50 values of actionable and non-actionable cell lines was only significant with $p<0.1$ in the available data sets.

Our results demonstrate that in addition to correctly predicting/confirming all known actionable mutations for their respective histotype for which sufficient data was available in the cell line data bases, protein profile discriminability between presence or absence of actionable is predictive of drug response in cell line data across cancers ($p=0.048$, Table 1).

The following section describes our drug response validation for each studied actionable gene in more detail:

For ERBB2, we evaluated response data of the drugs lapatinib, neratinib and cuduc-101. ERBB2 is known to be actionable in gastric cancer and breast cancer. For our analysis, a sufficient number of cell lines were available with response data for lapatinib and neratinib for breast cancer (BRCA), gastric cancer (STAD), lung adenocarcinoma (LUAD) and pancreatic ductal adenocarcinoma (PAAD). The analysis of protein profiles of TCGA cases did show protein profile discriminability between actionable and non-actionable cases for BRCA, STAD and LUAD. Also a cross-cancer effect between BRCA, STAD and LUAD was found. Protein profiles for PAAD were not

discriminable. For breast and gastric cancer, lapatinib and neratinib ic50 values differ significantly between actionable and non-actionable cell lines (p-Values: lapatinib: BRCA p=0.013, STAD 0.075, neratinib: BRCA p=0.046, p=STAD 0.049). Also, ic50 values below 1 are overrepresented among cases with actionable mutations for both tumor types and drugs (p-Values: lapatinib: BRCA p=0.076, STAD p=0.048, neratinib: BRCA p=0.012, STAD p=0.055). For PAAD no differences in ic50 values and no overrepresentation of sensitive cell lines were found. For PAAD and ERBB2 our analysis had shown no difference between protein profiles of actionable and non-actionable TCGA cases. For lung adenocarcinoma differences in ic50 values between actionable and non-actionable cell lines were found for lapatinib (p=0.04) and neratinib (p=0.093) and an overrepresentation of sensitive cell lines within actionable cell lines was found for lapatinib (p=0.061). For cudc-101 there were enough LUAD and BRCA cell lines tested to perform our analysis. For breast cancer, no differences in ic50 values or overrepresentation was found (p=0.68, p=0.452). For lung adenocarcinomas ic50 values were different between actionable and non-actionable cell lines (p=0.037) and there were significantly more sensitive cell lines among the actionable cell lines (p=0.035). These are the most important results of our drug response analysis, as for TCGA data we saw a cross-cancer effect for ERBB2 between the actionable tumor types breast and stomach cancer and lung adenocarcinomas. Our results imply that this cross-cancer effect goes along with similar therapy response fur lung cancer which is not yet actionable for ERBB2.

For BRAF V600 mutations response data on the drug dabrafenib was available and there were enough skin cancer cell lines with actionable mutation (43) to perform our analysis (17 cell lines without actionable mutation available). For TCGA protein data we saw protein profile discriminability and skin cancer is the actionable tumor type for these mutations. A difference in ic50 values between cell lines with and without actionable mutation was found (p=0.005) within the cell line data and sensitive cell lines were more frequent within the cell lines with actionable mutation (p=0.018).

For EGFR mutations targeted by gefitinib the TCGA data showed protein discriminability between actionable and non-actionable cases for lung cancer, which is the actionable tumor type for this drug. Cell line drug response data showed a significant number of sensitive cell lines (p=0.037) within lung cancer cell lines with actionable mutation.

FGFR1 amplification is actionable for small cell lung cancer, for which TCGA protein profiles were discriminable. Cell line data showed a significant number of sensitive cell lines (p=0.082) for this tumor type among actionable cell lines. Also, for colon cancer enough cell lines with mutation were available for test, yet no difference in sensitivity was found (p=0.73). For colon cancer TCGA protein profiles have been not discriminable also.

KRAS/NARS mutations are actionable for skin cancer, thyroid cancer and colorectal cancer. For thyroid and skin cancer, we discovered differences in protein profiles between actionable and non-actionable cases in the TCGA data. For the drug selumentinib we found a significant number of sensitive and actionable skin cancer cell lines (p=0.085). For this drug we also found significant differences in ic50 values between actionable and non- actionable cell lines for lung adenocarcinoma (p=0.021), for which TCGA protein profiles have been discriminable. For gastric, colon and uterine cancer no difference in drug sensitivity was found (p=0.93, p=0.22, p=0.44).

TCGA protein profiles have been discriminable for uterine cancer but not for gastric and colon cancer.

MET amplifications are actionable for kidney renal cancer and sarcomas. For both tumor types TCGA protein profiles of actionable and non-actionable cases have been discriminable. For lung adenocarcinoma there were differences in ic50 values between actionable and non-actionable cases (p=0.026) for the drug cabozantinib. For gastric cancer a significant number of cell lines was sensitive to cabozantinib (p=0.038), whereas TCGA protein profiles had not been discriminability for gastric cancer.

For PIK3CA no drug for which sensitivity data was available showed a sensitivity of breast cancers cell lines, although PIK3CA is actionable for breast cancer. For the drug ic-87114 bladder cancer cell lines showed a significant difference of ic50 values, comparing cell lines with and without actionable mutation (p=0.042) and a significant number of actionable cell lines had low sensitivity values (p=0.059). TCGA protein profiles of actionable and non-actionable bladder cancer cases have been not discriminable for this gene. There was no difference in drug response to ic-87114 for head and neck squamous carcinoma cell lines. TCGA protein profiles for this tumor type have been not discriminable between PIK3CA actionable and non-actionable cases.

A complete list of results is given in Additional file5.

Materials and Methods: Cross-cancer similarities for different gene sets

While TCGA provides comprehensive mutational profiling data covering all genes, RPPA protein expression data from TCGA/TCPA is limited to a set of less than 200 proteins, which may introduce a bias when comparing cross-cancer similarities between genomic and proteomic data. To address this issue, we first, reperformed our similarity analysis for only those genes with corresponding proteins in the TCPA/RPPA data. While certain quantitative differences obviously exist between using all genes and only those corresponding to the available proteins, the overall pattern of substantial cross-cancer similarities is consistent (Figure S3, left). Second, to evaluate if grouping genes by their functional effects or pathway membership makes a difference and would produce more histotype-specific pattern as for the proteins, we reperformed the analysis after assigning genes to their pathways based on the Broad institute's MSigDB gene set c6 (oncogenic signaling). Also for this setting, no qualitative change is seen and even on this (functional) gene set level, genetic information shows pronounced cross-cancer similarities not observed on the level of proteins (Figure S3, right).

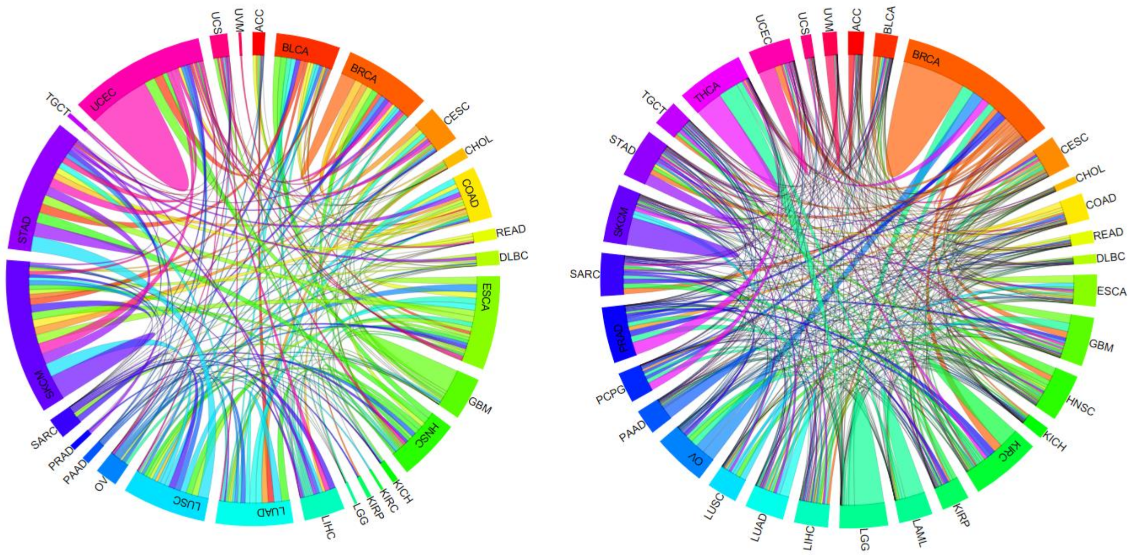

Figure S3: Mutational cross-cancer similarities for reduced sets of genes. While our main analysis relies on all available genes, we reperformed the analysis to test for biases due limited numbers of proteins. Results show that the overall patterns are consistent also for reduced numbers of genes corresponding to available proteins and functional gene sets: (Left) Chord diagrams show the cross-cancer similarities only for those genes for which the corresponding protein data is available and (right) based on genes aggregated by MSigDB gene set c6 -oncogenic signaling- by computing for each case the closest molecular neighbor among all other tumors. Chords connecting two histological tumor types indicate the number of tumors of a certain cancer that are more similar to tumors of the other type than to their own, indicating a disagreement of molecular and histological type. Hill-like structures, on the other hand, indicate the amount of cases where molecular and histological class are identical.

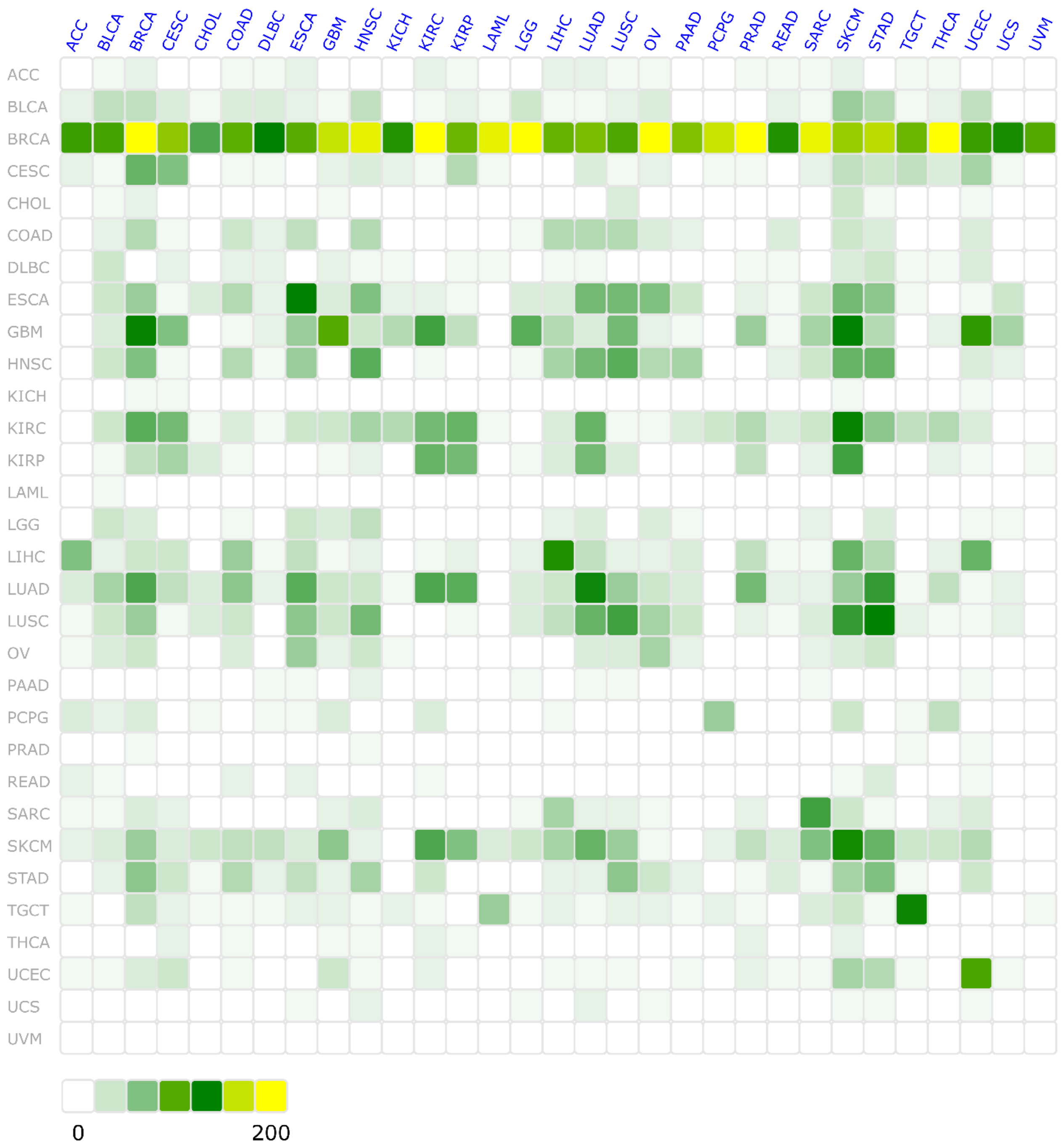

Figure S3a: Mutational cross-cancer similarities for reduced sets of genes. Same information as shown in the left part of Figure S3 formatted as heatmap (mutational similarity based on genes corresponding to available protein data – only cases with more than two mutated genes included). Number of cases of a tumor type (row) with most similar other case from another tumor type (column).

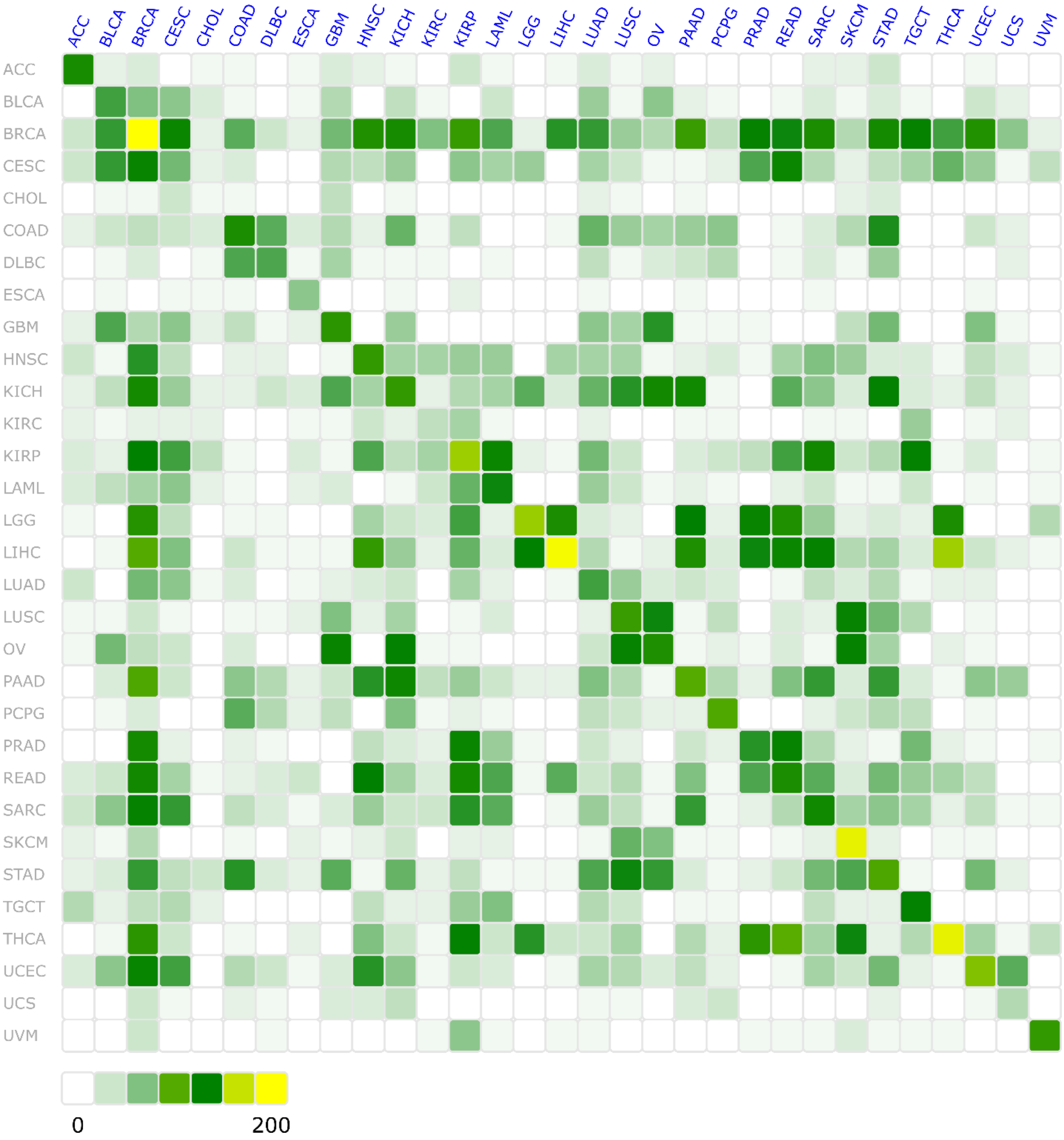

Figure S3b: Mutational cross-cancer similarities for reduced sets of genes. Same information as shown in the right part of supplementary Figure S3 formatted as heatmap (similarity analysis based on MSigDB gene set c6). Number of cases of a tumor type (row) with most similar other case from another tumor type (column).

Finally, we found another indication that different levels of molecular information show different degrees of cross-cancer similarities by performing our analysis also on DNA methylation and gene expression data. Here, our analysis shows pronounced histotype-specificity for global DNA methylation data similar to what we observe for proteins. Gene expression (RNAseq) data takes an intermediate position with less cross-cancer similarities than methylation and protein data, but

more histotype-specificity than somatic mutations and copy number variation. These data cover all genes and are therefore unbiased (Figure S4).

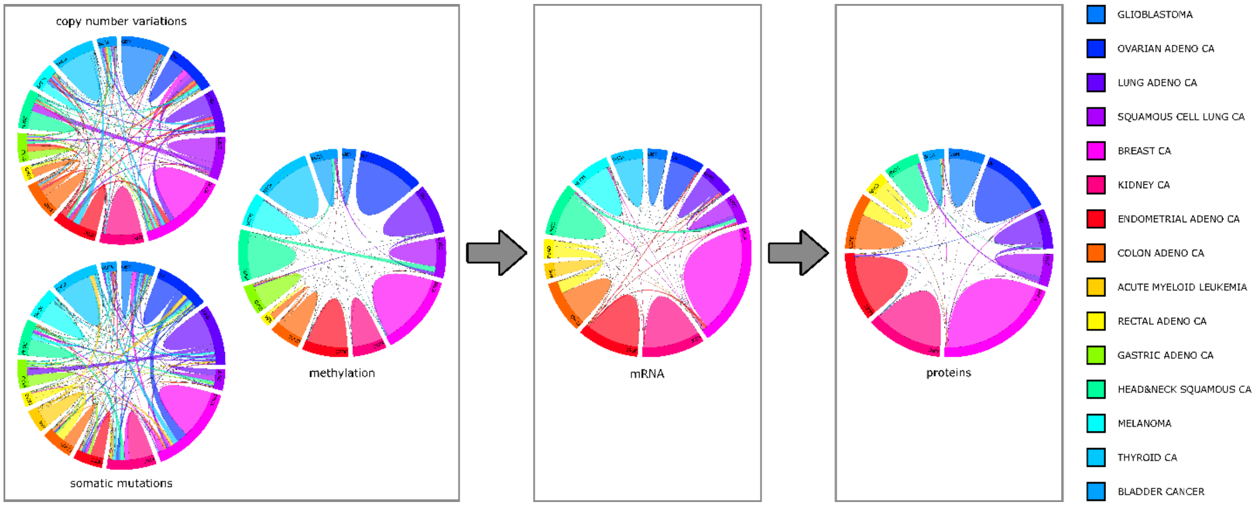

Figure S4: Cross-cancer similarities based on somatic mutations, copy number variation, methylation, mRNA and proteins visualized with Chord diagrams (compare Fig. 1). The analysis shows pronounced cross-cancer similarities (multiple connection chords) for CNVs and somatic mutations (left) whereas methylation and protein profiles are largely histotype-specific. Gene expression (mRNA) takes an intermediate position in terms of relationship of molecular profiles with histotypes.

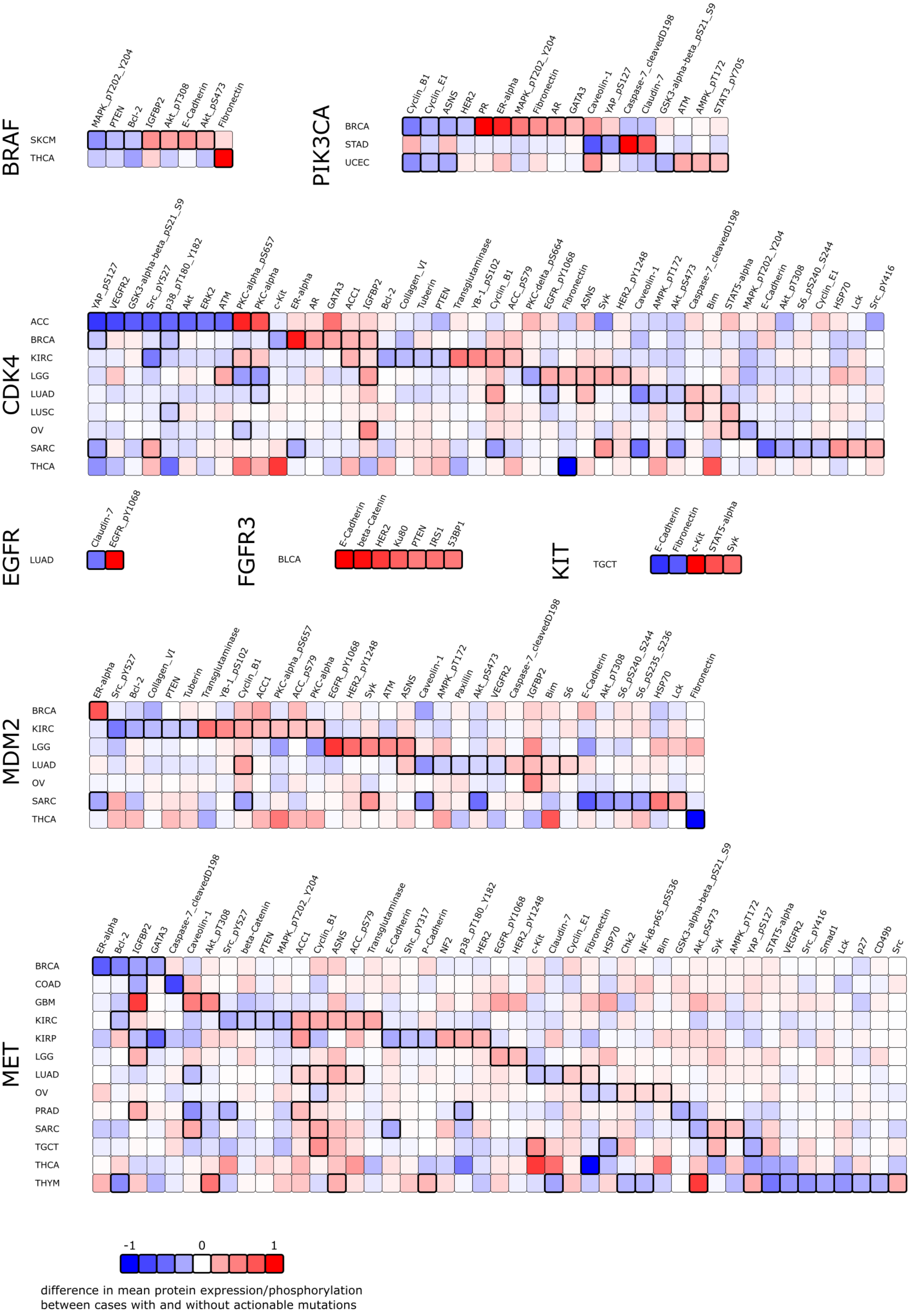
BRAF
SKCM
THCA
MAPK_pT202_Y204
PTEN
Bcl-2
IGFBP2
Akt_pT308
E-Cadherin
Akt_pS473
Fibronectin
PIK3CA
BRCA
STAD
UCEC
Cyclin_B1
Cyclin_E1
ASNS
HER2
PR
ER-alpha
MAPK_pT202_Y204
Fibronectin
AR
GATA3
Caveolin-1
YAP_pS127
Caspase-7_cleavedD198
Claudin-7
GSK3-alpha-beta_pS21_S9
ATM
AMPK_pT172
STAT3_pY705
CDK4
ACC
BRCA
KIRC
LGG
LUAD
LUSC
OV
SARC
THCA
YAP_pS127
VEGFR2
GSK3-alpha-beta_pS21_S9
Src_pY527
p38_pT180_Y182
Akt
ERK2
ATM
PKC-alpha_pS657
PKC-alpha
c-Kit
ER-alpha
AR
GATA3
ACC1
IGFBP2
Bcl-2
Collagen_VI
Tuberin
PTEN
Transglutaminase
YB-1_pS102
Cyclin_B1
ACC_pS79
PKC-delta_pS664
EGFR_pY1068
Fibronectin
ASNS
Syk
HER2_pY1248
Caveolin-1
AMPK_pT172
Akt_pS473
Caspase-7_cleavedD198
Bim
STAT5-alpha
MAPK_pT202_Y204
E-Cadherin
Akt_pT308
S6_pS240_S244
Cyclin_E1
HSP70
Lck
Src_pY416
EGFR
LUAD
Claudin-7
EGFR_pY1068
FGFR3
BLCA
E-Cadherin
beta-Catenin
HER2
Ku80
PTEN
IRS1
53BP1
KIT
TGCT
E-Cadherin
Fibronectin
c-Kit
STAT5-alpha
Syk
MDM2
BRCA
KIRC
LGG
LUAD
OV
SARC
THCA
ER-alpha
Src_pY527
Bcl-2
Collagen_VI
PTEN
Tuberin
Transglutaminase
YB-1_pS102
Cyclin_B1
ACC1
PKC-alpha_pS657
ACC_pS79
PKC-alpha
EGFR_pY1068
HER2_pY1248
Syk
ATM
ASNS
Caveolin-1
AMPK_pT172
Paxillin
Akt_pS473
VEGFR2
Caspase-7_cleavedD198
IGFBP2
Bim
S6
E-Cadherin
Akt_pT308
S6_pS240_S244
S6_pS235_S236
HSP70
Lck
Fibronectin
MET
BRCA
COAD
GBM
KIRC
KIRP
LGG
LUAD
OV
PRAD
SARC
TGCT
THCA
THYM
ER-alpha
Bcl-2
IGFBP2
GATA3
Caspase-7_cleavedD198
Caveolin-1
Akt_pT308
Src_pY527
beta-Catenin
PTEN
MAPK_pT202_Y204
ACC1
Cyclin_B1
ASNS
ACC_pS79
Transglutaminase
E-Cadherin
Shc_pY317
P-Cadherin
NF2
p38_pT180_Y182
HER2
EGFR_pY1068
HER2_pY1248
c-Kit
Claudin-7
Cyclin_E1
Fibronectin
HSP70
Chk2
NF-kB-p65_pS536
Bim
GSK3-alpha-beta_pS21_S9
Akt_pS473
Syk
AMPK_pT172
YAP_pS127
STAT5-alpha
VEGFR2
Src_pY416
Smad1
Lck
p27
CD49b
Src
-1
0
1
difference in mean protein expression/phosphorylation
between cases with and without actionable mutations

Figure S5: Characteristic proteins for actionable genes: Identification of proteins characteristic of cross-cancer effects. Four out of the 12 studied actionable genes were found to have cross-cancer effects on the level of available protein profiles (compare main manuscript Figure 4). Proteins were evaluated for their role in discriminating between wildtype and mutated actionable gene. Bold border outlines indicate statistically significantly characteristic proteins for the given histological tumor type (rows). Brackets indicate the pairs of histological tumor types for which the actionable mutations show same directional effects (indicated by an overlap of characteristic proteins with same directional change). Histotype names with cross-cancer effect are colored blue

Table S1: Batches removed due to possible batch effect

Tumor Type	Batches to remove	Sample count
BLCA	128	18
COAD	28,33,45,36	69
GBM	4	9
KIRP	209	6
LUAD	166,160	53
PRAD	308,285,91,268,429	135
READ	67,43,42,133,102	65
SARC	310	26
UCEC	137	12
		sum: 393

Supplementary .xls File "Pathway analysis" (Additional file 2)

Details on the results of the Ciriello pathway analysis.

Supplementary .csv File "Results" (Additional file 3)

A detailed listing of all results.

Supplementary .xls File "Actionable genes" (Additional file 4)

A table containing all information on evaluated actionable genes.

Supplementary .xls File "Cell line analysis" (Additional file 5)

Tables containing results on cell line data analysis.
